# Evaluating replicability in microbiome data

**DOI:** 10.1093/biostatistics/kxab048

**Published:** 2021-12-30

**Authors:** David S Clausen, Amy D Willis

**Affiliations:** Department of Biostatistics, University of Washington, Hans Rosling Center for Population Health, Box 351617, Seattle, WA 98195-1617, USA; Department of Biostatistics, University of Washington, Hans Rosling Center for Population Health, Box 351617, Seattle, WA 98195-1617, USA

**Keywords:** Classification, Clustering, Genomics, High-dimensional statistics, Measurement error, Reproducibility

## Abstract

High-throughput sequencing is widely used to study microbial communities. However, choice of laboratory protocol is known to affect the resulting microbiome data, which has an unquantified impact on many comparisons between communities of scientific interest. We propose a novel approach to evaluating replicability in high-dimensional data and apply it to assess the cross-laboratory replicability of signals in microbiome data using the Microbiome Quality Control Project data set. We learn distinctions between samples as measured by a single laboratory and evaluate whether the same distinctions hold in data produced by other laboratories. While most sequencing laboratories can consistently distinguish between samples (median correct classification 87% on genus-level proportion data), these distinctions frequently fail to hold in data from other laboratories (median correct classification 55% across laboratory on genus-level proportion data). As *identical samples* processed by different laboratories generate substantively different quantitative results, we conclude that 16S sequencing does not reliably resolve differences in human microbiome samples. However, because we observe greater replicability under certain data transformations, our results inform the analysis of microbiome data.

## 1. Introduction

Microscopic organisms are known to play a critical role in human and environmental health (Thompson *and others,* 2017; Huttenhower *and others,* 2012; Cho and Blaser, 2012). Microorganism communities, known as microbiomes, are considerably less stable than human genetic elements (Huttenhower *and others,* 2012; Cho and Blaser, 2012), and for this reason disease interventions that manipulate the host’s microbiome are being actively pursued by researchers in gastrointestinal health, oral health, and reproductive health (Huttenhower *and others,* 2012; Cho and Blaser, 2012; Kelly *and others,* 2016). The quality of the data collected by these researchers is the subject of investigation in this article.

There are many different experimental approaches to studying a host-associated microbiome. One of the most widely employed approaches is to sequence a hypervariable region of the 16S rRNA gene. The data that results from a 16S sequencing experiment are the number of times each 16S sequence variant $j$ was observed in each sample $i$, which we call $W_{ij}$. Many steps are involved in generating a taxon abundance table $\{W_{ij}\}$ from specimens, including sample storage, DNA extraction, sequencing, and raw data processing (bioinformatics), and all of these steps are known to impact the resulting profiles (Pollock *and others*, 2018; Hugerth and Andersson, 2017; Sinha *and others,* 2017; Gibbons *and others,* 2018).

Our objective in this article is to assess replicability, the degree to which independent teams of researchers studying the same phenomenon are able to obtain congruent results (Nosek and Errington, 2020), in 16S data. A related concept, *reproducibility*—which concerns transparent documentation of data analysis enabling outside researchers, given original study data, to rerun an analysis and obtain identical results—is not the focus of this article.

In particular, we are interested in the impact of measurement protocols on replicability, a topic that is both understudied and scientifically consequential: if one research team cannot replicate the findings of another, it is difficult to assess the evidence for the finding. Accordingly, we investigate how consistent observed differences between samples are in measurements taken by different sequencing laboratories. Differences between samples are a focus of research in human microbiome science; a typical study might inspect whether a measured feature differs across groups of subjects defined by disease status. In view of the large diversity of features investigated in human microbiome studies, we do *not* examine the replicability of any particular feature (e.g., a particular diversity index or a specific microbial strain), but instead are interested in the *overall* inter-laboratory similarity of measured distinctions at common scales of comparison.

Because of our emphasis on the replicability of *differences between samples* (as opposed to, e.g., a meta-analysis of effect sizes), we analyzed data from the Microbiome Quality Control (MBQC) Project. This data set contains 22 extremely dissimilar specimens: 2 are low-complexity artificial communities, 2 are drawn from a bioreactor, and 18 are from human subjects. Of the 18 human specimens, 9 are male, 5 are female, with donor sex unknown for the remainder. The age range of human specimens spans 2 years to 70 years, and approximately one-third of samples are from ICU cases, one-third are pre- or postsurgery, and one-third are healthy.

The disparate sources of samples in this data set should lead to observable differences in 16S sequencing data. Indeed, we are able to identify distinctions between samples that hold in individual sequencing laboratories (low within-laboratory technical variation). However, we also find very different distinctions between samples across laboratories (high cross-laboratory variation). Hence, while within-laboratory technical variability is low enough to allow differentiation of samples, observed distinctions between samples are not robust to between-laboratory technical variation, suggesting that measurement error may mask or bias between-subject and between-group comparisons in human 16S studies.

## 2. Data and model

### 2.1. Data set

The MBQC Project was established by the MBQC Consortium to “comprehensively evaluate methods for measuring the human microbiome” (Sinha *and others,* 2015, pp. 1–2). A major objective was to quantitatively compare the results of 16S sequencing as implemented by multiple research groups on identical samples. To this end, Sinha *and others* (2017) distributed identical microbiome sample sets comprising samples from 22 unique specimens to 15 participating sequencing laboratories that were blinded to the samples’ labels. Each laboratory prepared and analyzed sample sets according to a sequencing protocol of their choice. Raw data from each sequencing laboratory were then sent to nine bioinformatics laboratories, which were blind to sequencing laboratory identity as well as specimen origin of samples. Bioinformatics laboratories processed the data according to an analysis protocol of their choosing. The taxon abundance tables from each sequencing-bioinformatics combination were submitted in standardized format.

We denote the taxon abundance data collected by Sinha *and others* (2017)$\{W_{ijklm}\}$, where $i \in \{1, \dots, I\}$ indexes the specimen from which a sample was taken, and $j \in \{1, \dots, J\}$ indexes the operational taxonomic unit (OTU) to which the count is attributed. We further index taxon abundance data according to the sequencing laboratory $k \in \{1,\dots, K\}$ and bioinformatics laboratory $l \in \{1, \dots, L\}$ that generated it. $m \in \{ 1, \dots, M_{ikl}\}$ indexes replicate measurements on specimen $i$ within sequencing laboratory $k$$\times$ bioinformatics laboratory $l$. We note in particular the distinction between “specimen” and “sample” here, which we will maintain throughout this article: a “specimen” is a unique source of genetic material, portions of which may be extracted for sequencing; a “sample” is such an extracted portion—that is, it is the unit of sequencing.

### 2.2. Model and model evaluation

We wish to evaluate the widely held belief that “each protocol will have a set of biases that affect all samples equally” (Sinha *and others,* 2017, p. 1081), and in particular that on this basis measurement error may safely be disregarded in microbiome analyses. To this end, we propose a statistical model for the MBQC data to formalize and explore the implications of this claim. Let $\rho_{ij}$ be the true, unknown relative abundance of taxon $j$ in specimen $i$, and $\vec{W}_{i\cdot klm} = (W_{i1klm}$$\ldots, W_{iJklm})$ be the observed counts from all taxa in specimen $i$ by sequencing laboratory $k$, and dry laboratory $l$. For simplicity, we omit further reference to bioinformatics laboratories $l$, which our analysis treats as replicates (see Section 3.3), and we let $M_{ik}$ represent the number of replicate observations on specimen $i$ reported for laboratory $k$. (Although all sequencing laboratories were sent identical sample sets, some laboratories were sent multiple sets.) We represent transformed sequencing count data $\vec{W}_{i\cdot km}$ as a sum of transformed true relative abundances $\vec{\rho}_{i \cdot }$ and an error term $\vec{\epsilon}_{\Psi i \cdot km}$:

(2.1) \begin{equation*}\label{error_model_eq} \vec{\Psi}(\vec{W}_{i\cdot km}) = \vec{\Psi}(\vec{\rho}_{i\cdot}) + \vec{\epsilon}_{\Psi i \cdot km},\end{equation*}

where $\Psi$ is some scale-invariant transformation of the count data (e.g., relative abundance or centered log-ratio; we consider only scale-invariant transformations because $\sum_{j = 1}^J W_{ij}$ is an artifact of the sequencing experiment). We formalize the idea of protocol biases affecting specimens equally via assumptions on the expectation of the error term $\vec{\epsilon}_{\Psi i \cdot km}$:

(2.2) \begin{equation*}\label{error_expectation} \mathbb{E}[\vec{\epsilon}_{\Psi i \cdot km}] = \vec{c}_{\Psi \cdot km}.\end{equation*}

That is, $\vec{\Psi}(\vec{W}_{i\cdot km})$ is a biased estimate of $\vec{\Psi}(\vec{\rho}_{i\cdot})$, with bias $\vec{c}_{\Psi \cdot k}$ constant across samples $i$ but potentially differing across taxa $j$. (Note that bias of $\vec{\Psi}(\vec{W}_{i\cdot km})$ for $\vec{\Psi}(\vec{\rho}_{i\cdot})$ includes bias resulting from nonlinearity of $\vec{\Psi}$.) If we lift the first moment assumption on $\vec{\epsilon}_{\Psi i \cdot km}$, the model above is completely general; in particular, we note that, with or without first-moment conditions, this model makes no assumptions on similarity of error distributions across taxa.

We do not know the true abundances $\vec{\rho}_{i \cdot}$ for the MBQC data, nor in most 16S experiments. However, because we are interested in evaluating replicability, we do not attempt to estimate the $\vec{\rho}_{i \cdot}$’s. Instead, we propose a statistical machine learning approach to evaluate a key implication of model (2.1)–(2.2), with errors modeled as independent across samples.

We first note that under the above model, the expected difference between transformed observed abundances and transformed true abundances depends on sequencing laboratory $k$ and the chosen transformation $\Psi$, but not on the sample composition $i$. Consequently, differences in transformed measurements between specimens are invariant in expectation across laboratory: $\mathbb{E}[\Psi(\vec{W}_{i\cdot k}) - \Psi(\vec{W}_{i'\cdot k})] = \mathbb{E}[\Psi(\vec{W}_{i\cdot k'}) - \Psi(\vec{W}_{i'\cdot k'})]$ (for simplicity, we suppress the replicate index $m$ here).

Therefore, we can evaluate the model in the absence of knowing $\vec{\rho}_{i \cdot}$ by assessing if observed between-specimen structure is conserved across laboratories.

To do this, we split data as follows. As each sequencing laboratory received multiple physical samples of each of the 22 unique specimens included in this study, we divide data produced by sample. We assign each sample sequenced by a given laboratory either to the training or the test set for that laboratory, with each sample assigned to training or test sets with equal probability and each training and test set containing at least one sample from each unique specimen. All bioinformatics results reported for a given sample share the set assignment of the sample.

This procedure produces, for each sequencing laboratory $k$, a training set $W^{\text{train}}_k$ and a test set $W^{\text{test}}_{k}$. Separately on each of these sets we calculate “sample-centered” transformed measurements (Section 3.2), which we call $\Psi'(W)$. On each training set $W^{\text{train}}_k$, we attempt to find a function $\phi_k: range(\Psi') \rightarrow \{1, \ldots, I\}$ such that expected misclassification error

(2.3) \begin{align*} \label{eq:misclass} \frac{1}{\sum_{i = 1}^I M_{ik}\sum_{i = 1}^{I} \sum_{m = 1}^{M_{ik}} \mathbb{E}\bigg[\mathbf{1}_{\big[ \phi_k( \Psi'(W_{i \cdot km})) \neq i \big]}\bigg]} \end{align*}

is small. In other words, $\phi_k$ is a classifier that identifies specimen label $i$ and was trained on data from sequencing laboratory $k$ (Section 3.3). We then use $\phi_k$ to predict specimen labels on *every* sample-centered test set $W^{\text{test}}_k$: both for the laboratory that the classifier was trained on, and all other laboratories.

**Fig. 1.**
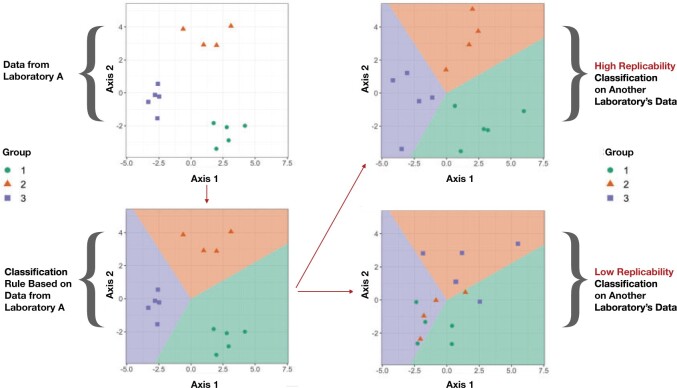
Performance of a classifier trained on centered transformed taxon abundances in Laboratory A indicates the degree to which similar between-specimen structure is able to be resolved in data from another sequencing laboratory.

Finally, we analyze the magnitude and patterns in the classifiers’ misclassification rates, including which transformations better preserve distinctions between samples; at which taxonomic resolution we observe highest replicability; whether some laboratories detect more replicable distinctions between samples than others; and the difference in classifier performance within- versus across-laboratories. Figure 1 illustrates how our classifier-based approach distinguishes between structure-preserving and structure-distorting measurement error across sequencing laboratories.

In short, our approach allows us to evaluate how similar measurement error is across sequencing laboratories in terms of our ability to distinguish specimens after we have removed a form of bias that is irrelevant to estimating differences between specimens. When the distribution of measurement error in laboratories $k$ and $k'$ is identical up to a constant (i.e., up to $\vec{c}_{\Psi \cdot k} - \vec{c}_{\Psi \cdot k'}$), we expect classifiers to perform equally well in either laboratory. When measurement error distributions are not equal up to a mean shift, our approach provides an empirical measure of the degree of difference between them. We express this result in terms of “centered” error terms $\epsilon_{\Psi i \cdot km} - \mathbb{E}[\epsilon_{\Psi i \cdot km}]$, where expectation is taken over the joint distribution of class (specimen) labels $i$ and class-and-laboratory-specific error terms $\epsilon_{\Psi i \cdot km}$ (note that by design the distribution of class labels $i$ is identical across laboratories). In particular, if we let $Q_k$ denote the joint distribution of centered error terms $\epsilon_{\Psi i \cdot km} - \mathbb{E}[\epsilon_{\Psi i \cdot km}]$ and the class labels $i$ in laboratory $k$, and analogously for laboratory $k'$ with $Q_{k'}$, we have for any classification rule $\phi(X)$ that $|\mathbb{E}_{Q_k}[\mathbf{1}_{[\phi(X) \neq I]}] - \mathbb{E}_{Q_{k'}}[\mathbf{1}_{[\phi(X) \neq I]}]| \leq d_{\text{TV}}(Q_{k},Q_{k'})$. That is, the absolute value of the difference in expected misclassification rates lower bounds the total variation distance between $Q_k$ and $Q_{k'}$. In practice, we center by sample averages since $\mathbb{E}[\epsilon_{\Psi i \cdot km}]$ is not known, and so the inequality holds up to $\mathcal{O}(1/\sqrt{n_k \wedge n_{k'}})$ term, if $n_k$ is the number of samples sequenced by laboratory $k$, and similarly for $n_{k'}$ and laboratory $k'$. (See Appendix A of the Supplementary material available at *Biostatistics* online for a formal statement and proof).

## 3. Methods

### 3.1. Data sources and treatment of missing data

We considered OTU count data published by Sinha *and others* (2017) in Nature Biotechnology (Supplementary Data set 6 available at https://www.nature.com/articles/nbt.3981). As explained in Sinha* and others*, data from bioinformatics laboratories BL-3 and BL-5 were excluded from Supplementary Data set 6 as these laboratories did not report counts in standardized format. We stored available data as a table with columns representing variables and rows observations using the data.table package (version 1.12.6) (Dowle and Srinivasan, 2019) in R (version 3.6.1) (R Core Team, 2019). We then filtered out all rows for which sequencing lab, bioinformatics lab, or specimen was listed as missing or unknown, as well as negative controls and pre-extracted DNA samples. As reported in Sinha *and others* (2017, p. 1079), some bioinformatics groups did not report results for samples with read counts below a given threshold. This induced missingness in some combinations of sequencing lab, dry lab, and specimen. To avoid confounding bioinformatics and sequencing laboratory effects, as well as to ensure that sufficient data were available to train and validate classifiers in each sequencing laboratory we considered, we limited our analysis to a subset of sequencing and bioinformatics laboratories with sufficient completeness in each combination of sequencing and bioinformatics laboratory. Details of the procedure used to select this subset of laboratories are available in Appendix B of the Supplementary material available at *Biostatistics* online.

### 3.2. Data transformations

Each laboratory provided taxon abundance tables with 16S sequence variants $j$ attributed to operational taxonomic units (OTUs), a unit based on observed 16S gene sequence similarity. OTUs can be organized according to a taxonomy comprising (from finest to broadest) species, genus, family, order, class, and phylum. To assess degree of replicability at finer or broader aggregations, we trained classifiers for every level of taxonomic aggregation.

For every taxonomic aggregation level, we consider two transformations $\Psi$ that are commonly used in microbiome analyses. We consider the proportion transformation

$$\begin{align*}
\Psi_1: \mathbb{R}^{J} \rightarrow \mathbb{S}^{J-1} ; \Psi_1(\vec{W}_{i \cdot k}) &= \left( \frac{W_{i1k}}{\sum_{j = 1}^J W_{ijk}}, \dots, \frac{W_{iJk}}{\sum_{j = 1}^J W_{ijk}} \right)\!,\end{align*}$$

and the centered log-ratio transformation

$$\begin{align*}
\Psi_2: \mathbb{R}^{J} \rightarrow \mathbb{R}^J ; \Psi_2(\vec{W}_{i \cdot k}) = \left( \text{log}\frac{W_{i1k}}{\left(\prod_{j = 1}^J W_{ijk}\right)^{1/J}}, \dots, \text{log}\frac{W_{iJk}}{\left(\prod_{j = 1}^J W_{ijk}\right)^{1/J}} \right)\!\label{abcd}.\end{align*}$$

Note that the centered log-ratio transform is only defined when $W_{ijk}>0$ for all $j$, so in practice, zero counts in taxon tables are frequently replaced by a small positive “pseudocount” prior to transformation (Quinn *and others,* 2019). In accordance with this practice, we replaced all zero counts with a pseudocount of 1 before transformation. We investigated the sensitivity of our results to the choice of pseudocount and found negligible differences in classifier performance. We note that adding a pseudocount breaks the scale invariance of $\Psi_2$.

To ensure that classifiers learn features of the data that reflect between-specimen structure and do not depend on $\vec{c}_{\Psi \cdot k}$, for transformations $\Psi_1$ and $\Psi_2$ we center measurements on samples from specimens $1$ through $I$ from every test or training set by subtracting $\frac{ 1}{I}\sum_{i = 1}^I \frac{1}{M_{ik}^{\text{set}}}\sum_{m' = 1}^{M_{ik}^{\text{set}}}\Psi(\vec{W}_{i\cdot k m'})$ from each observation, where $M_{ik}^{\text{set}}$ is the number of replicate measurements for specimen $i$ in the sample set (either a training or test set). We performed this centering to ensure that the resulting centered quantities have expectation that does not depend on $\vec{c}_{\Psi \cdot k}$:

(3.4) \begin{align*} &\phantom{=} \Psi(\vec{W}_{i\cdot km}) - \frac{ 1}{I}\sum_{i = 1}^I \frac{1}{M_{ik}^{\text{set}}}\sum_{m' = 1}^{M_{ik}^{\text{set}}}\Psi(\vec{W}_{i\cdot k m'}) \\\end{align*}

(3.5) \begin{align*} & = \big[ \Psi(\vec{\rho}_{i\cdot}) + \epsilon_{\Psi i \cdot km} \big] - \frac{1}{I}\sum_{i' = 1}^I \frac{1}{M_{ik}^{\text{set}}}\sum_{m'= 1}^{{M_{ik}^{\text{set}}}} \big[ \Psi(\vec{\rho}_{i'\cdot}) + \epsilon_{\Psi i' \cdot km'} \big] \\ \end{align*}

(3.6) \begin{align*} &= \big[ \Psi(\vec{\rho}_{i\cdot}) - \frac{1}{I}\sum_{i' = 1}^I \Psi(\vec{\rho}_{i'\cdot})\big] + \big[\epsilon_{\Psi i \cdot km} - \frac{1}{I}\sum_{i' = 1}^I \frac{1}{M_{ik}^{\text{set}}}\sum_{m'= 1}^{M_{ik}^{\text{set}}} \epsilon_{\Psi,i' \cdot km'} \big] := \Psi'(\vec{\rho}_{i\cdot}) + \epsilon'_{\Psi i \cdot km},\end{align*}

where $\Psi'(\vec{\rho}_{i\cdot})$ and $\epsilon'_{\Psi i \cdot km}$ are defined to be the first and second bracketed terms of the LHS of (3.6), respectively. We additionally} define $\Psi'(\vec{W}_{i\cdot km})$ calculated on either a test or training from sequencing laboratory $k$ : $\Psi'(\vec{W}_{i\cdot km}) := \Psi(\vec{W}_{i\cdot km}) - \frac{ 1}{I}\sum_{i = 1}^I \frac{1}{{M_{ik}^{\text{set}}}}\sum_{m' = 1}^{{M_{ik}^{\text{set}}}} \Psi(\vec{W}_{i\cdot k m'})$.

If the model given in Section 2.2 holds, we have

(3.7) \begin{align*} \mathbb{E} \epsilon'_{\Psi i \cdot km} &:= \mathbb{E} \Big[ \big[\epsilon_{\Psi i \cdot km} - \frac{1}{I}\sum_{i' = 1}^I \frac{1}{{M_{ik}^{\text{set}}}}\sum_{m'= 1}^{{M_{ik}^{\text{set}}}} \epsilon_{\Psi,i' \cdot km'} \big]\Big] \\\end{align*}

(3.8) \begin{align*} &= c_{\psi \cdot k} - \frac{1}{I}\sum_{i' = 1}^I \frac{1}{{M_{ik}^{\text{set}}}}\sum_{m'= 1}^{{M_{ik}^{\text{set}}}} c_{\psi \cdot k} = \vec{\mathbf{0.}}\end{align*}

Hence, the centered transformed measurements are equal to centered transformed true relative abundances plus a mean-zero error under model (2.1)–(2.2).

### 3.3. Training and validation of boosted tree and elastic net classifiers

To train and validate classifier performance, we first assign samples sequenced by each sequencing laboratory to either the test or the training set for that laboratory. For each unique specimen, samples taken from that specimen are assigned to training or test sets with equal probability, and each training and test set contains at least one sample from each unique specimen. All bioinformatics results reported for a given sample share the set assignment of the sample.

On each training set, we trained boosted regression tree classifiers with R package xgboost (version 0.90.0.2) (Chen *and others,* 2019) and elastic net classifiers with R package glmnet (version 2.0.18) (Friedman *and others,* 2010) to classify samples according to which specimen they were taken from. We then predicted on the test set to obtain estimates of within-laboratory misclassification rates for each classifier. We also predicted specimen label on test sets for sequencing laboratories not used to train the classifier to obtain estimates of cross-laboratory misclassification rates.

For both boosted tree and elastic net classifiers, we selected parameters via 10-fold cross-validation on training sets. Details of parameter selection are provided in Appendix B of the Supplementary material available at *Biostatistics* online. For each combination of $k$, $\Psi$ and taxonomic level, a distinct classifier was trained.

We briefly note here that overfitting (i.e., selection of high-variance classifiers) is a common concern in high-dimensional settings. For this reason, we chose classifiers (boosted tree and elastic net) and a training procedure (10-fold cross-validation) with some robustness to this problem (Bühlmann *and others,* 2007; Friedman *and others,* 2004; Zou and Hastie, 2005). Moreover, we constructed training and test sets so that classifiers are trained and evaluated on measurements taken on *completely disjoint* sets of samples, so misclassification error on test sets is unbiased for population misclassification error if errors $\epsilon_{\psi i\cdot km}$ are independent across samples $m$. Conversely, if dependence of errors $\epsilon_{\psi i\cdot km}$ within sequencing laboratory drives lower within- than across-laboratory misclassification error, this indicates that observed distinctions between samples depend on sequencing laboratory—the very phenomenon we aim to investigate in this manuscript.

To investigate conservation of observed distinctions among more disparate samples, we conducted an additional analysis focusing on specimen type rather than specimen. For this analysis, we categorized the 22 unique specimens analyzed by the MBQC into four broad types: human (18 specimens); chemostat (2 specimens); artificial fecal community (1 specimen); artificial oral community (1 specimen). We performed sample centering using these types, trained classifiers with type labels, and evaluated classifier performance predicting specimen type on held-out test sets from each laboratory. In all other respects, we observed the same protocol as in our primary analysis.

All misclassification results shown in Section 4 are based on the testing sets (no misclassification rates for training data are shown).

Code to reproduce the analysis is available at github.com/statdivlab/mbqc_supplementary.

## 4. Results

### 4.1. Proportion-scale data

**Fig. 2.**
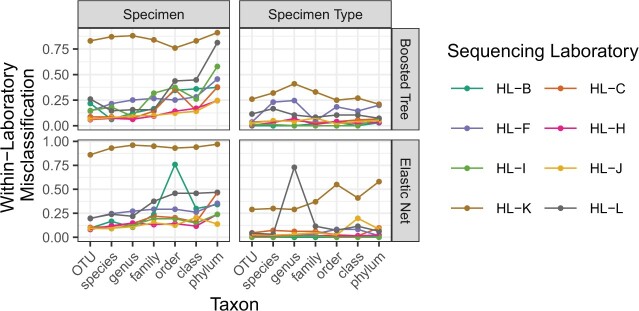
Within-laboratory misclassification for boosted tree and elastic net classifiers predicting specimen or specimen type on proportion data plotted against level of taxonomic aggregation. Color indicates the laboratory that the classifier was trained and evaluated on.

We first examine performance of each classifier on held-out test data from the laboratory on which the classifier was trained. Within-laboratory, out-of-sample predictions provide a baseline against which to compare cross-laboratory performance. Within-laboratory misclassification rates on proportion-scale data ($\Psi_1$) is shown in Figure 2.

Within sequencing laboratory, signals distinguishing specimen types exhibit generally low misclassification error: median 5% (IQR 2–11%) for boosted tree signals and 3% (IQR 1– 8%) for elastic net. This low misclassification is likely due both to the relatively large biological differences between specimen types (human, oral mock, fecal mock, or chemostat) and to the composition of sample sets sent to sequencing laboratories, which were composed of $\sim$75% human fecal samples on average.

Specimen misclassification is generally higher than specimen type misclassification on within-laboratory replication, with median specimen misclassification 22% (IQR 12–38%) for boosted tree classifiers. This higher misclassification reflects both the increased biological similarity between specimens (versus specimen types) and the relatively even distribution of specimens across samples: no specimen accounts for more than 6% of samples sent to a sequencing laboratory.

Additionally, within-laboratory replicability of between-specimen signals appears to decrease as taxonomy coarsens. Median within-laboratory misclassification of boosted tree classifiers is 15% (IQR 8–23%) on OTU-level data, rising to 42% (IQR 34–64%) on phylum data. The corresponding figures for elastic net classifiers are 10% (IQR 9–20%) and 35% (IQR 24–47%), respectively.

Spikes in misclassification of elastic net classifiers on HL-B and HL-L (for specimen and specimen type classification, respectively) are explored in greater detail in Appendix E of the Supplementary material available at *Biostatistics* online. In short, they likely result from three sources: relatively high within-laboratory technical variation in HL-L; probable splitting of (unlabeled) batches in HL-B across training and test sets; and the sensitivity of the elastic net to the distribution of measurement error under the sum-to-one constraint imposed at the proportion scale.

**Fig. 3.**
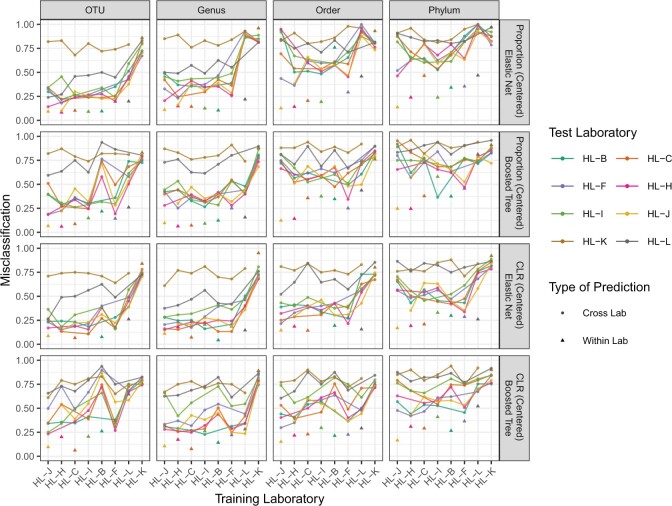
Within-laboratory (solid triangles) and between-laboratory (lines) misclassification for boosted tree and elastic net classifiers predicting specimen on sample-centered proportion (first two rows) and centered log-ratio (third and fourth rows) data plotted against level of taxonomic aggregation. Color indicates the laboratory whose data the classifier was trained on.

To investigate the degree to which between-specimen structure was conserved across sequencing laboratory, we used each of the classifiers trained on proportion data to predict specimen using proportion data from every other laboratory. The performance of these classifiers in terms of misclassification on OTU, genus, order, and phylum data is summarized in Figure 3. See Appendix I of the Supplementary material available at *Biostatistics* online for results for specimen and specimen type at all taxonomic levels.

Between-specimen signals learned from proportion data replicate far less strongly across- than within-sequencing laboratory. Median within-laboratory misclassification for boosted tree classifiers is 22% (IQR 12–38%), compared to 65% (IQR 45–78%) across-laboratory.

Within- and cross-laboratory misclassification both generally increase with coarsening taxonomy. For the boosted tree classifiers, median misclassification on OTU-level data is 15% within-laboratory (IQR 8–23%) versus 55% cross-laboratory (IQR 30–75%). On phylum-level data, these figures are 35% (IQR 24–7%) and 82% (IQR 66–88%), respectively. On phylum-level data, elastic net classifiers perform similarly, attaining 30% (IQR 24–42%) median within-laboratory and 71% (IQR 55–%) cross-laboratory misclassification. On OTU-level data, elastic net classifiers marginally outperform boosted tree classifiers, with median within-laboratory misclassification 10% (IQR 9–20%) and median cross-laboratory misclassification 34% (IQR 24–61%).

### 4.2. Log-ratio transformed data

16S data is also frequently analyzed after a log-ratio transformation, an approach from the compositional data literature (see, e.g., Aitchison, 1982). In this section, we examine replicability of between-specimen signals on the centered log-ratio scale.

As with proportion-scale data, between-specimen signals learned from centered log-ratio data replicate more strongly within than across sequencing laboratory (Figure 3). For boosted tree classifiers, median within-laboratory misclassification is 24% (IQR 17–31%), in contrast to 60% (IQR 42–75%) cross-laboratory misclassification.

Within- and cross-laboratory misclassification both generally increase with coarsening taxonomy (Figure 3). For the boosted tree classifiers, median within-laboratory misclassification on OTU-level data is 23% (IQR 17–44%) versus 66% (IQR 41–75%) for cross-laboratory misclassification. On phylum data, these figures are 33% (IQR 28–44%) and 74% (IQR 58–81%), respectively.

### 4.3. Summary of findings

**Fig. 4.**
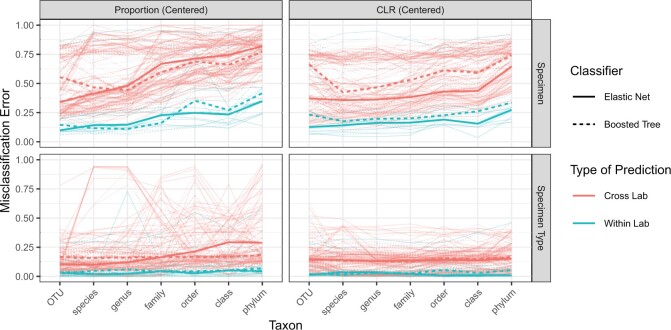
The within-laboratory (aqua) and cross-laboratory (red) misclassification for centered proportion and centered log ratio data for classifiers predicting both specimen and specimen type. The misclassification rate is shown for boosted tree (solid lines) and elastic net (dotted lines) classifiers for every combination of laboratories (thin lines) and is also summarized as a median across laboratory combinations (thick lines).

Figure 4 summarizes our findings, showing the misclassification error for each pair of training and test laboratories rendered as a thin line segment. For each combination of transformation and classification task (specimen vs. specimen type), median within- and cross-laboratory misclassification is plotted against taxon as a bold line.

Regardless of classifier, for each transformation and at every level of taxonomic aggregation we considered, median *within-laboratory* specimen misclassification is substantially lower than median *cross-laboratory* specimen misclassification. That is, *sequencing laboratories find distinctions between samples that cannot be replicated in other laboratories.* Whether this occurs because some sequencing laboratories report spurious reads (e.g., contamination) or because laboratories are differentially able to measure various features of microbial communities is not clear from this analysis. Both sources of error likely contribute to the patterns observed in this analysis, and the scale and level of taxonomic aggregation at which data are considered may determine the relative importance of each source.

For each transformation and classifier we considered, within- and cross-laboratory specimen misclassification generally increase with increasing level of taxonomic aggregation, although the strength of this trend varies by transformation. That is, *replicability decreases with increasing taxonomic aggregation.* This result most likely reflects information lost as a result of aggregation. Additionally, the pattern of cross-laboratory misclassification suggests that measurement error is not mitigated by taxonomic aggregation. That is, our findings suggest that between-phylum signals are generally less replicable than signals at finer taxonomic levels, possibly because the effects of measurement error are more similar in closely related taxa than in distantly related taxa. We therefore recommend analysis on data at the genus level or finer.

Within-laboratory misclassification was generally lower than cross-laboratory misclassification under every data transformation we considered. With respect to both within- and cross-laboratory misclassification, however, proportion data were particularly unreliable, with specimen type classifiers frequently failing to outperform simply classifying all samples as human. Measurement error due to differential detection efficiency and contamination are nonlinear on this scale, which may explain for this behavior. Hence, we expect statistical inference on means of 16S proportion data to be particularly sensitive to measurement error.

### 4.4. Supplemental analyses

We perform several analyses to investigate the sensitivity of our analysis to various modeling choices. Appendix D of the Supplementary material available at *Biostatistics* online explores whether we can find a subgroup of sequencing laboratories across which between-specimen signals replicate well. In Appendix F of the Supplementary material available at *Biostatistics* online, we assess the influence of pooling results from multiple bioinformatics laboratories via a sensitivity analysis in which we fit and predict from classifiers using only data reported by bioinformatics laboratories individually. In Appendix G of the Supplementary material available at *Biostatistics* online, we fit classifiers to centered and rescaled sequencing data to investigate whether laboratory- and taxon-specific scalings could explain our results, and we additionally provide descriptive plots of measured abundance across four major phyla in fresh human specimens by sequencing laboratory and specimen. Appendix H of the Supplementary material available at *Biostatistics* online reports results for within- and cross-laboratory performance of classifiers trained on presence–absence data, another commonly used transformation of sequencing read data.

## 5. Discussion

Our objective in this article was to assess the replicability in 16S data, and to this end, we focused on the inter-laboratory similarity of measured distinctions between specimens. Focusing on between-specimen distinctions allowed us to assess evidence for claim that “each protocol will have a set of biases that affect all samples equally” (Sinha *and others,* 2017, p. 1081) and that on this basis between-group comparisons can be resolved in the presence of measurement error. Our analysis also estimates a lower bound on the total variation distance between the residual measurement error distributions across sequencing laboratories after a laboratory-specific bias term has been canceled.

We found that replication of between-specimen signals is stronger within- than across-sequencing laboratory, even when laboratories analyze identical specimens. This is consistent with the recent work of Wirbel *and others* (2021), who found substantially lower cross-study than within-study performance of classifiers trained to identify disease states on the basis of data from a single study. Notably, cross-study predictive performance improved when training data was augmented with external study data. Both these results and our own suggest that *laboratory-specific measurement error may mask or distort between-group comparisons of 16S data.*

While we did not directly test the applicability of any given model connecting sample composition $\rho_{i \cdot}$ to taxon abundance tables $W_{i \cdot}$, our findings were broadly consistent with the multiplicative detection effect model of McLaren *and others* (2019). This model predicts that between-laboratory replicability should be greater on centered log-ratio data, where, given sufficiently deep sequencing, multiplicative distortions should cancel in our analysis. In centered log-ratio data, this canceling should occur primarily at fine levels of taxonomy, as the multiplicative detection effects described in McLaren *and others* (2019) properly apply to microbes at the strain level; at higher taxonomic levels, strains varying in degree of detectability will be grouped together, and the model of McLaren *and others* (2019) will fit less well. We do in fact observe these patterns (in particular, compare classifier performance on centered log ratio versus proportion data with and without sample centering, as reported in Appendix I of the Supplementary material available at *Biostatistics* online), suggesting that differential detection of certain taxa by protocols may drive some of the measurement error we observe.

### 5.1. Limitations and interpretation of results

In this analysis, we set out to assess conservation of between-specimen signals within and across sequencing laboratories. As the form of these signals was not known a priori, we used flexible classifiers to learn between-specimen distinctions and then assessed replicability in terms of misclassification error of classifiers trained on training sets from each sequencing laboratory. In order to train classifiers, we pooled data across bioinformatics laboratories for use as replicates. While these decisions allowed us to illustrate the impact of measurement error on between-specimen signals, they each introduce limitations into our analysis.

While our analytical approach allowed us to flexibly learn distinctions between specimens, this flexibility prevented our analysis from highlighting any specific set of taxa as contributing to replication failure. This reflects our goal of evaluating the replicability of measured between-specimen distinctions, though it renders the applicability of our findings to any particular experimental result more difficult to determine.

In addition, while we chose to examine 16S data under two transformations commonly used in microbiome analyses, microbiome studies employ a wide range of analytic techniques. These include approaches borrowed from the RNA-seq literature such as limma, an empirical Bayes method that can incorporate observation reweighting via an estimated mean–variance relationship (Law *and others,* 2014), as well as DESeq2 and edgeR, which attempt to account for technical variation via normalizations either applied directly to data as transformations or included as terms in a model (Robinson *and others,* 2010; Love *and others,* 2014), Though we did not explore the replicability of observed differences between specimens after application of normalizations commonly used in RNA-seq analyses, we note that RNA-seq (as well as microbiome) methods have previously been found not to control type-1 error in microbiome data (Hawinkel *and others,* 2019). In a similar vein, we were unable to address the totality of statistical methods developed specifically for 16S data in our analysis, and it is unclear how our results will generalize across methods.

We chose to use taxon abundance data reported by participating bioinformatics laboratories rather than reprocess raw read data. Accordingly, our results, particularly for fine taxonomic levels, do not reflect recent developments in bioinformatics protocol, such as the ability to identify exact 16S sequence variants (Callahan *and others,* 2016, 2017). Furthermore, as we estimate and validate signals over bioinformatics replicates, we estimate misclassification error averaged over bioinformatics laboratories. We investigate the sensitivity of our analysis to pooling of bioinformatics in Appendix F of the Supplementary material available at *Biostatistics* online.

The conclusions we can draw from this analysis are also limited by the MBQC data set. As the MBQC Project was not a designed experiment with respect to laboratory protocol (laboratories chose their own protocols), laboratory effects may confound any observed protocol effects. Additionally, the distribution of some protocol variables is highly unbalanced, rendering inference imprecise, even in the absence of confounding. For example, among the laboratories we included in our analysis, six out of eight used the same 16S primer, with the other two each using a distinct primer. For these reasons, we chose not to estimate effects due to protocol variables directly.

Additionally, the population of laboratories included in the MBQC may not represent the global population of laboratories that generate microbiome data. For instance, laboratories participating in the MBQC study may have differed from the typical laboratory conducting 16S sequencing in terms of funding and academic profile. Furthermore, participating laboratories knew that they were participating, which may have changed their behavior (referred to as the Hawthorne effect). Relatedly, bioinformatics laboratories discarded samples as quality control, and it is unclear if bioinformatics teams working in collaboration with sequencing teams would discard samples as readily. For these reasons, we chose to present descriptive summaries of classifier performance rather than perform inference on misclassification rates.

The generalizability of our analysis is also limited by the range of specimens included in the MBQC. As all human specimens included in this study were fecal samples, our results are most relevant to studies of the human gut microbiome. In a similar vein, as the true composition of these specimens is unknown, we were only able to assess consistency of measurements across laboratories. Therefore, we are not able to recommend any particular sequencing protocol for estimating true sample composition.

## 6. Conclusion

In the past two decades, failures of replication in quantitative disciplines ranging from social science to biomedical research have attracted considerable scientific and public attention (Loken and Gelman, 2017; Ioannidis *and others,* 2001; Simmons *and others,* 2011). Concern over replication failures is well-founded: if independent groups of researchers cannot replicate scientific findings, this calls into question to what extent the published literature reflects objective reality.

In this article, we evaluated the replicability of high-dimensional microbiome data obtained from 16S sequencing. By analyzing a data set wherein identical samples were distributed to different sequencing laboratories, we demonstrated that measurement error in 16S studies degrades the replicability of measured distinctions between specimens. The degree of nonreplicability depends on the data transformation and level of taxonomic aggregation used in analysis. We derived this result by training flexible classifiers to identify specimens using data from individual sequencing laboratories and comparing their performance on held-out test sets taken from the laboratories on which they were trained versus on test sets from other laboratories. On species-level data, the classifiers *correctly* classified a median of 64% and 56% of specimens predicting on, respectively, centered log ratio and proportion data based on test data from a *different* laboratory than the laboratory that generated the training data. These figures were substantially lower than the corresponding figures for classification on out-of-sample test data from the *same* laboratory used to train classifiers, 84% and 87%.

In general, we observed larger misclassification errors at coarser levels of taxonomy. For example, on phylum-level centered log-ratio data, classifiers correctly classified a median of 71% of specimens within laboratory, but only 30% across laboratory. In addition, we found that even when observed within-laboratory technical variation was low, replication of between-specimen structure suffered in cross-laboratory comparisons.

These results suggest that measurement error in 16S studies may mask or distort distinctions between specimens. Hence, in our view 16S profiles are best understood as providing a noisy picture of microbial communities, and caution should be exercised when interpreting the results of a 16S analysis. Accordingly, we advocate for the independent validation of conclusions drawn from 16S sequencing (Minot and Willis, 2020).

The findings of this manuscript highlight the need for further research in the characterization of measurement error in sequencing of microbial communities. For example, McLaren *and others* (2019) recently demonstrated that observed profiles of simple communities differ from the true profiles by taxon-specific multiplicative factors that can be attributed to components of the sequencing workflow (e.g., extraction and amplification). The model of McLaren *and others* (2019) may partially explain some of our findings. If the model of McLaren *and others* (2019) applied perfectly to the MBQC data, we would expect similar levels of cross-laboratory and within-laboratory misclassification in CLR-transformed species-level abundance data. However, we observe a median correct classification of 64% and 84% across- and within-laboratory misclassification, respectively, a discrepancy highlighting the need for further characterization of measurement error in human 16S data.

More positively, our findings suggest that certain analyses may be more robust to measurement error than others. In particular, *our results point to analyses at fine taxonomies on a log-ratio scale as more likely to replicate*, although this pattern did not hold in every cross-laboratory comparison in our analysis. This has a number of practical implications for many different types of microbiome analysis, including inference (log-ratio based models may be more robust to measurement error than relative-abundance models), and visualization (ordination using Aitchison distance may be more appropriate than using other measures of dissimilarity).

Experimental techniques to study microbial communities continue to be developed, and whole-genome “shotgun” sequencing, long-read sequencing, microbial single-cell sequencing, and microbial transcriptomics are becoming increasingly prevalent approaches to surveying microbiomes. To our knowledge, a study where identical samples were distributed to data collection centers that use these alternative microbial community profiling techniques has not been performed. As our analysis indicates that within-laboratory consistency does not in general guarantee cross-laboratory consistency, we encourage microbial ecologists to consider the potential for cross-laboratory inconsistency in emerging experimental techniques until cross-laboratory consistency has been demonstrated.

## Supplementary Material

kxab048_Supplementary_Data
