# Supplementary material for: Evaluating replicability in microbiome data
Source: Biostatistics. 2021 Dec 30;23(4):1099–114. doi: 10.1093/biostatistics/kxab048 (PMC9566336; doi:10.1093/biostatistics/kxab048)
Supplement: kxab048_Supplementary_Data [file kxab048_supplementary_data.pdf]

Biostatistics (2021), **0**, 0, *pp.* 1–38
doi:10.1093/biostatistics/mbqc-biostatistics-supp-v2

Supplementary Information for Evaluating Replicability in Microbiome Data

Web Appendix A: Proofs

Lower bound for total variation distance

Consider an arbitrary measurable function $\phi : \mathbb{R}^J \to \mathcal{Z}$ where $\mathcal{Z} = \{1, \ldots, I\}$, and note that any classification rule on classes $1, \ldots, I$ based on data $X \subset \mathbb{R}^J$ can be expressed as such a function. Letting Q_k be the joint distribution of class labels $Z \in \mathcal{Z}$ and taxonomic covariates X (with sample space $\mathcal{X}$) in a sequencing laboratory k, we have, for the absolute value of the difference of expected misclassification error in laboratories k and k' of an arbitrary classification rule based on test function ϕ:

$$
\begin{aligned}
&|Q_k \mathbf{1}_{[\phi(X) \neq Z]} - Q_{k'} \mathbf{1}_{[\phi(X) \neq Z]}| \\
&\qquad = |Q_k(1 - \mathbf{1}_{[\phi(X)=Z]}) - Q_{k'}(1 - \mathbf{1}_{[\phi(X)=Z]})| \\
&\qquad = |Q_k \mathbf{1}_{[\phi(X)=Z]} - Q_{k'} \mathbf{1}_{[\phi(X)=Z]}| \\
&\qquad = |\int_{\{(x,z)\in\mathcal{X}\times\mathcal{Z}:\ \phi(x)=z\}} dQ_k(x,z) - \int_{\{(x,z)\in\mathcal{X}\times\mathcal{Z}:\ \phi(x)=z\}} dQ_{k'}(x,z)| \\
&\qquad \leqslant \sup_{A\subset\mathcal{X}\times\mathcal{Z}} |\int_A dQ_k(x,z) - \int_A dQ_{k'}(x,z)| \\
&\qquad = d_{\mathrm{TV}}(Q_k, Q_{k'})
\end{aligned}
$$

Therefore, letting the joint distribution of covariates (i.e., sample-centered transformed counts) and specimens in a test set in laboratory k follow Q_k and the corresponding data in laboratory k'

© The Author 2021. Published by Oxford University Press. All rights reserved. For permissions, please e-mail: journals.permissions@oup.com

follow $Q_{k'}$, the difference in misclassification error of an arbitrary classification rule in laboratories k and k' is bounded in expectation by the total variation distance between Q_k and $Q_{k'}$.

Web Appendix B: Supplementary Methods

Data Exclusions

To evaluate the consistency of the taxonomic profile of identical biological samples observed by each lab, we trained classifiers to predict the specimen number of each sample (details below). We considered samples from freeze-dried human stool, fresh human stool samples, chemostat and mock communities that were extracted at the laboratory at which they were sequenced.

As few combinations of wet lab and bioinformatics lab had complete data among non-pre-extracted samples (no aliquots excluded by bioinformatics), we adopted a relaxed standard of sufficient completeness. Under this standard, we considered any combination of a wet and a dry lab to have sufficiently complete data for inclusion in our analysis only if more than 75% of aliquots were available, and only if data for every specimen was reported.

The imbalance of bioinformatics laboratory data available across aliquots from sequencing laboratories may create confounding between bioinformatics effects and sequencing laboratory effects. To minimize the impact of this confounding on our results, which concern sequencing laboratory effects, we constructed a procedure to find a subset of the bioinformatics labs and sequencing labs described in Sinha *and others* (2017) containing no excluded combination. We excluded all sequencing laboratories for which no bioinformatics laboratory provided sufficiently complete data as defined above. We then exhaustively searched all combinations of sequencing laboratory and bioinformatics laboratory to find sets of sequencing laboratories that had sufficiently complete data across a common set of at least four bioinformatics laboratories. We set this minimum number of bioinformatics laboratories to ensure sufficient data within wet lab to train and evaluate classifiers on. We found two combinations of 8 sequencing laboratories that

met these criteria: HL-E and HL-I. We chose to use the combination containing HL-I as HL-I had more complete data than HL-E.

Treatment of Unclassified Reads

Some bioinformatics laboratories classified some reads from sequencing lab data as unclassified at various taxonomic levels. We chose to include such reads classified to an unknown taxon in our analysis. We regard both misclassification of a known organism as an unknown organism and heterogeneity across sequencing labs of organisms classified as unknown as forms of measurement error that impact the replicability of taxonomic profiling via 16S.

Classifier Training and Validation

We selected boosted regression tree classifier parameters for each combination of wet lab, classification task, and level of taxonomic aggregation. There are four parameters to select:

1. Proportion of observations sampled per boosting step (0.5 or 1)

2. Proportion of covariates used at each boosting step (0.25, 0.5, 0.75, or 1)

3. Learning rate

4. Number of boosting steps

For each combination of the first two parameters, we trained a classifier minimizing multinomial logistic loss starting at learning rate 0.1 for 10,000 boosting steps, or until cross-validated misclassification error had not improved for 500 boosting steps, whichever occurred first. We then retrained for up to 5 iterations, with learning rate decreased by $\sqrt{10}$ at each successive iteration. If at any iteration, optimal 10-fold cross-validated misclassification error occurred after at least 1000 boosting steps, following the rule of thumb proposed by Elith *and others* (2008), we ended

iteration over training rate. We also ended iteration early if a model reached cross-validated misclassification rate 0. We then compared all fitted boosted tree models and selected parameters corresponding to the model with the lowest 10-fold cross-validated misclassification error. We resolved ties in favor of whichever model took the greatest number of boosting steps to reach the lowest cross-validated misclassification error, and any further ties in favor of, in order, lowest learning rate, variable subsampling, and observation subsampling. We fit our boosted trees using the `R` package xgboost (Chen *and others*, 2019).

We also selected elastic net parameters for each combination of wet lab, classification task, and level of taxonomic aggregation. There are two elastic net parameters to select:

1. λ: penalty magnitude

2. $\alpha \in \{0, 0.1, 0.2, \ldots, 1\}$: mixing proportion between L_1 and L_2 penalties

For each value of α, we evaluated many values of λ via automatic penalty selection algorithm provided in the `cv.glmnet` function in `glmnet` (Friedman *and others*, 2010). We then selected α and λ according to minimum 10-fold cross-validated misclassification error, resolving ties in favor of values of α closer to 0.5, and resolving any further ties in favor of the lower value of α.

Web appendix C: Data Completeness

	HL−A	HL−B	HL−C	HL−D	HL−E	HL−F_1	HL−F_2	HL−H	HL−I	HL−J	HL−K	HL−L	HL−M	HL−N_1	HL−N_2
BL−1	35	106	97	0	106	100	51	159	0	265	53	53	15	26	27
BL−2	35	105	97	0	71	99	51	159	53	265	53	53	15	26	27
BL−3	0	105	97	0	105	95	48	159	0	259	53	0	15	26	27
BL−4	35	105	97	44	106	99	51	159	53	265	53	53	15	26	27
BL−6	35	105	97	44	71	99	51	159	53	265	53	53	15	26	27
BL−8	6	104	97	40	103	91	46	156	53	264	52	53	15	26	27
BL−9A	35	103	97	0	106	99	51	159	0	264	53	53	15	26	27
BL−9B	0	106	97	44	71	101	53	159	53	265	53	0	15	26	27

Fig. 1: The number of raw (i.e., not centrally extracted) aliquots reported for each combination of sequencing (columns) and bioinformatics (rows) laboratories. Raw aliquots were distributed in sets of 53, with some sequencing laboratories analyzing multiple sets.

	HL−A	HL−B	HL−C	HL−D	HL−E	HL−F_1	HL−F_2	HL−H	HL−I	HL−J	HL−K	HL−L	HL−M	HL−N_1	HL−N_2
BL−1	22	22	22	0	22	22	22	22	0	22	22	22	10	18	17
BL−2	22	22	22	0	22	22	22	22	22	22	22	22	10	18	17
BL−3	0	22	22	0	22	22	22	22	0	22	22	0	10	18	17
BL−4	22	22	22	21	22	22	22	22	22	22	22	22	10	18	17
BL−6	22	22	22	21	22	22	22	22	22	22	22	22	10	18	17
BL−8	5	22	22	20	22	22	21	22	22	22	22	22	10	18	17
BL−9A	22	22	22	0	22	22	22	22	0	22	22	22	10	18	17
BL−9B	0	22	22	21	22	22	22	22	22	22	22	0	10	18	17

Fig. 2: The number of unique specimens for which any data was reported in each combination of sequencing (columns) and bioinformatics (rows) laboratories. 22 unique specimens (excluding negative controls) were sent to each sequencing laboratory.

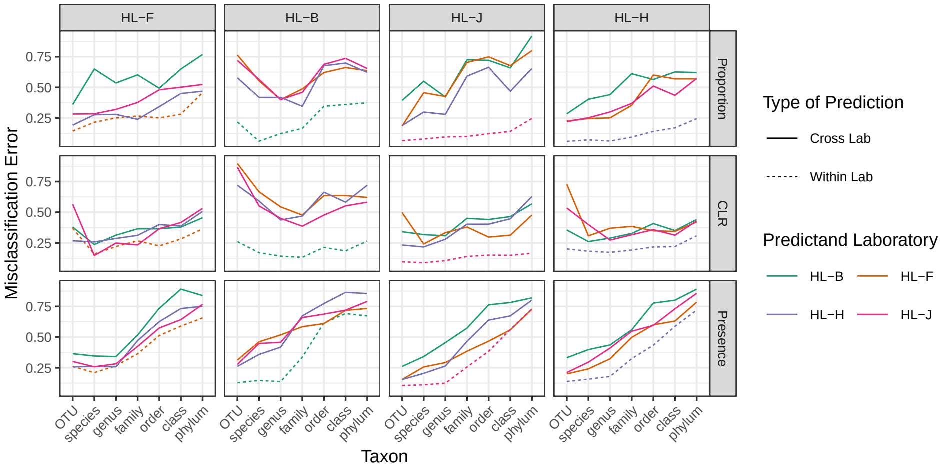

Fig. 3: Misclassification error of boosted tree specimen classifiers trained on centered proportion (top row), centered log ratio (middle row), and presence-absence (bottom row) data from four sequencing laboratories (columns). Across taxa (x-axis), within-laboratory misclassification (dotted lines) is typically lower than cross-laboratory misclassification (solid lines), but the size of this discrepancy depends on predictor laboratory, transformation, and taxonomic aggregation level.

Web appendix D: Supplementary Results

Replicability within subgroups of laboratories

Our primary analysis admits the possibility that between-specimen signals replicate well within some subgroup of sequencing laboratories. In figure 3 we present results for four sequencing laboratories with generally low within-laboratory misclassification. Figure 3 indicates that the gap between within-laboratory and cross-laboratory misclassification is generally largest for signals learned on proportion data. By contrast, between-specimen signals learned on centered log ratio data best replicate within and across laboratories in this comparison. At fine levels of taxonomy, signals learned on presence-absence data exhibit relatively strong within- and cross-laboratory replicability as well.

Alas, under no transformation nor level of taxonomic aggregation does within-laboratory replication well predict cross-laboratory replication. For instance, HL-B exhibits generally low

within-laboratory misclassification, but replicates relatively poorly on laboratories F, H, and J. Similarly, replication of a signal detected by one laboratory in another does not guarantee mutual replicability: centered log-ratio signals at fine levels of taxonomy in HL-F replicate fairly well in HL-B (24% versus 16% misclassification on species-level data from HL-B and HL-F, respectively), but signals learned on HL-B replicate relatively poorly in HL-F (66% versus 17% misclassification on species-level data from HL-F and HL-B, respectively). Taken together, these results suggest that low observed technical variation in a group of sequencing laboratories does not guarantee replicability of between-group signals detected by these laboratories. Moreover, replication of the results of one laboratory by another does not guarantee that the converse replication will hold.

Since our focus is assessing replicability, we chose not to model labs' protocol variables. However, the results presented in Figure 3 are consistent with known results about which protocol variables influence sample measurements. For instance, HL-F, HL-H, and HL-J generally replicate better on each other than on HL-B. HL-F, HL-H, and HL-J used extraction kits from the same manufacturer, while HL-B used a extraction kit from a different manufacturer. Although we cannot conclusively attribute this pattern to extraction protocol, it is in line with the large extraction effects reported in the microbiome literature (McLaren *and others*, 2019; Vebø *and others*, 2016).

Web Appendix E: Anomalous misclassification results in centered proportion data

In Figure 2 (main text), we observed a spike in the misclassification of elastic net classifiers trained on HL-B for specimen at the order level. We also observed a spike in the misclassification of elastic net classifiers trained on HL-L for specimen type at the genus level. In this section we investigate the source of these spikes.

In Figure 4 we see that the within-laboratory elastic net classifier trained on on HL-B centered proportion order-level data erroneously categorizes many aliquots as originating from sample 63. Figure 5, which shows average values of elastic net linear predictors (by specimen) in the HL-B centered proportion order-level training and test sets, suggests that poor performance on the HL-B test set is due to the influence of differing measurements in order Sphingomonadales between the training and test sets.

In Figure 6 we see that the within-laboratory elastic net classifier trained on on HL-L centered proportion genus-level data erroneously categorizes many aliquots as originating from samples 61 and 101. Figure 7, which shows average values of elastic net linear predictors (by specimen) in the HL-L centered proportion genus-level training and test sets, suggests that poor performance on the HL-L test set is due to measurements across various genera being discordant between the training and test sets.

Together these results show that discrepancies in the misclassification level of the elastic classifiers can be due to unusual test/train splits on a transformation of the data (i.e., proportion) at which error in any taxon propagates to all other taxa. However, we observed spikes in the misclassification error in only two of our elastic net classifiers. This gives us confidence that, in general, our classifiers are picking up distinctions between samples based on the data from the training laboratory, and not random noise.

	sample10	sample101	sample102	sample103	sample104	sample11	sample12	sample13	sample14	sample2	sample3	sample4	sample55	sample56	sample57	sample59	sample6	sample61	sample63	sample64	sample8	sample9
sample10	43	0	0	3	0	0	0	0	3	0	0	0	1	8	0	0	0	0	8	0	0	0
sample101	0	57	0	0	0	0	0	0	0	0	0	0	0	11	0	0	1	0	23	0	0	0
sample102	0	0	9	0	0	0	0	0	4	0	0	0	0	7	0	0	0	0	78	0	0	2
sample103	4	0	1	69	0	0	0	0	0	0	0	0	0	12	0	0	0	0	10	0	0	3
sample104	0	0	0	0	84	0	0	0	0	0	0	0	0	8	0	0	0	0	4	0	0	0
sample11	3	0	0	0	0	15	0	0	3	0	0	0	0	12	24	0	1	0	33	4	0	0
sample12	4	0	2	2	0	0	4	0	12	3	0	0	0	8	0	0	4	0	15	4	0	0
sample13	0	0	0	0	4	0	0	41	0	0	2	6	3	8	1	0	0	0	3	0	0	0
sample14	13	1	0	0	0	0	0	0	29	1	0	0	0	8	0	0	1	4	11	0	0	0
sample2	0	3	0	4	0	0	0	0	4	56	0	0	16	8	0	0	0	0	4	4	0	0
sample3	0	5	0	0	0	4	0	0	4	0	29	0	0	9	0	0	1	0	13	3	0	0
sample4	0	2	0	0	0	0	0	0	0	4	0	0	3	11	0	0	12	0	36	0	0	0
sample55	6	0	2	0	0	0	0	4	1	0	0	0	6	6	0	0	1	0	78	0	0	0
sample56	0	0	1	0	0	0	0	0	0	0	0	1	0	8	0	0	0	0	58	0	0	0
sample57	8	0	0	0	0	0	0	0	3	0	0	0	0	8	4	0	0	0	41	0	0	0
sample59	0	9	0	0	0	0	0	0	0	0	8	0	0	4	0	11	16	0	4	48	0	0
sample6	0	0	0	1	0	0	0	0	0	0	31	0	0	9	0	0	9	0	14	0	0	0
sample61	0	0	0	4	0	0	0	0	0	0	0	1	0	9	0	0	0	0	54	0	0	0
sample63	0	4	0	0	0	0	0	0	2	0	0	0	0	8	0	0	1	0	53	0	0	0
sample64	0	0	0	0	0	0	0	0	0	0	0	0	0	4	0	0	0	0	0	60	0	0
sample8	1	2	0	0	0	0	1	0	8	1	0	0	1	12	0	0	2	0	18	4	44	1
sample9	0	0	2	2	0	0	4	0	15	1	0	0	0	4	0	0	4	0	15	8	8	0

Fig. 4: A confusion matrix for within-laboratory elastic net classification on HL-B centered proportion order-level data. True labels are given as row names, and predicted labels are given in column names. This classifier erroneously categorizes many aliquots as originating from sample 63.

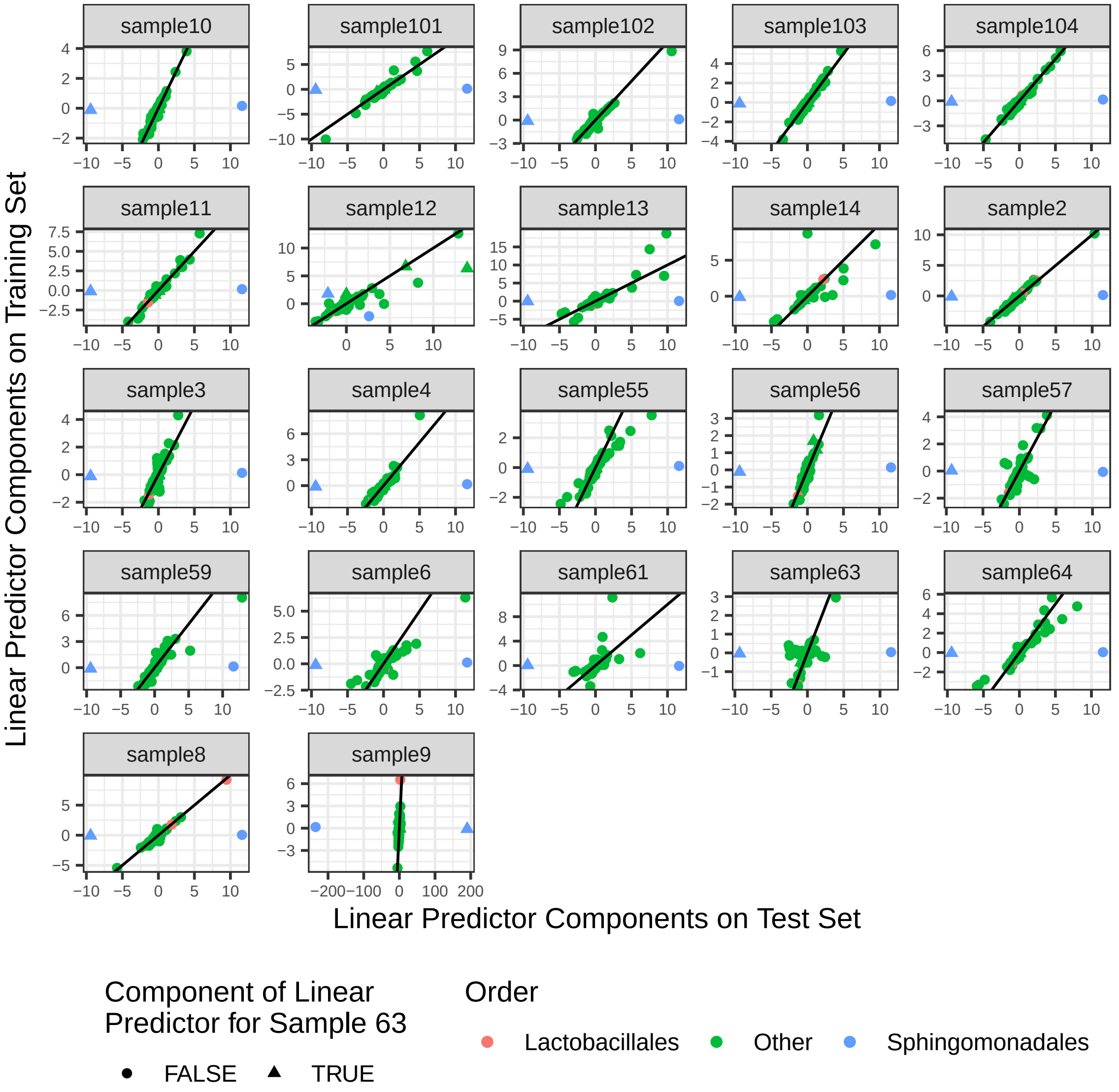

Fig. 5: Mean values of linear predictors (centered proportion multiplied by elastic net coefficients) by specimen for elastic net classifier trained on centered proportion order-level specimen data. The line x = y is shown in black. The linear predictor for order Sphingomonadales is generally large in aliquots in the training set, but not in the test set.

HL-L Genus Within-Lab Performance

	sample10	sample101	sample102	sample103	sample104	sample11	sample12	sample13	sample14	sample2	sample3	sample4	sample55	sample56	sample57	sample59	sample6	sample61	sample63	sample64	sample8	sample9
sample10	4	27	0	0	0	0	0	0	0	0	4	0	2	0	0	0	0	27	2	0	0	0
sample101	0	4	0	0	0	2	0	0	0	0	7	78	1	0	0	0	0	0	0	0	0	0
sample102	0	10	4	0	0	0	0	0	0	0	86	0	0	0	0	0	0	0	0	0	0	0
sample103	0	32	0	4	0	0	0	0	0	0	0	0	0	0	0	0	0	63	0	0	0	0
sample104	0	0	0	0	3	0	0	0	0	0	0	93	0	0	0	0	0	0	0	0	0	0
sample11	0	12	0	0	0	56	0	0	0	0	23	0	1	3	0	0	0	0	0	0	0	0
sample12	0	21	0	0	0	1	4	0	0	0	6	3	0	0	3	0	0	19	0	0	0	1
sample13	0	21	0	0	0	0	0	2	0	0	0	4	3	0	0	0	15	23	0	0	0	0
sample14	0	26	0	0	0	0	0	0	7	0	0	0	0	0	0	0	0	35	0	0	0	0
sample2	0	38	1	0	0	2	0	0	0	4	5	0	0	0	0	0	0	47	0	0	2	0
sample3	0	4	0	0	0	1	0	0	0	0	55	4	0	0	0	0	0	4	0	0	0	0
sample4	0	4	0	0	0	2	0	0	0	0	56	0	0	0	0	0	6	0	0	0	0	0
sample55	1	44	0	0	0	14	0	0	0	0	0	0	6	0	0	0	0	39	0	0	0	0
sample56	0	18	0	0	0	0	0	0	0	0	24	0	0	4	0	0	0	20	2	0	0	0
sample57	0	16	0	0	0	17	0	0	0	0	0	0	0	0	4	0	0	27	0	0	0	0
sample59	0	37	0	0	0	0	0	0	0	0	0	0	0	0	0	21	4	32	4	0	0	2
sample6	0	5	0	0	0	0	0	0	0	0	51	0	0	0	0	0	8	0	0	0	0	0
sample61	0	19	0	0	0	12	0	0	0	0	0	0	0	0	0	0	0	37	0	0	0	0
sample63	0	20	0	0	0	12	0	0	2	0	0	0	0	4	0	0	0	20	7	0	0	3
sample64	0	31	0	0	0	0	0	0	0	0	0	0	0	0	0	0	0	27	4	2	0	0
sample8	0	75	0	0	0	0	0	0	0	0	0	1	0	0	0	0	0	15	0	0	4	0
sample9	0	15	0	0	0	1	0	4	0	0	3	0	0	0	0	0	0	40	0	0	0	0

Fig. 6: A confusion matrix for within-laboratory elastic net classification on HL-L centered proportion genus-level data. True labels are given as row names, and predicted labels are given in column names. This classifier erroneously categorizes many aliquots as originating from samples 61 and 101.

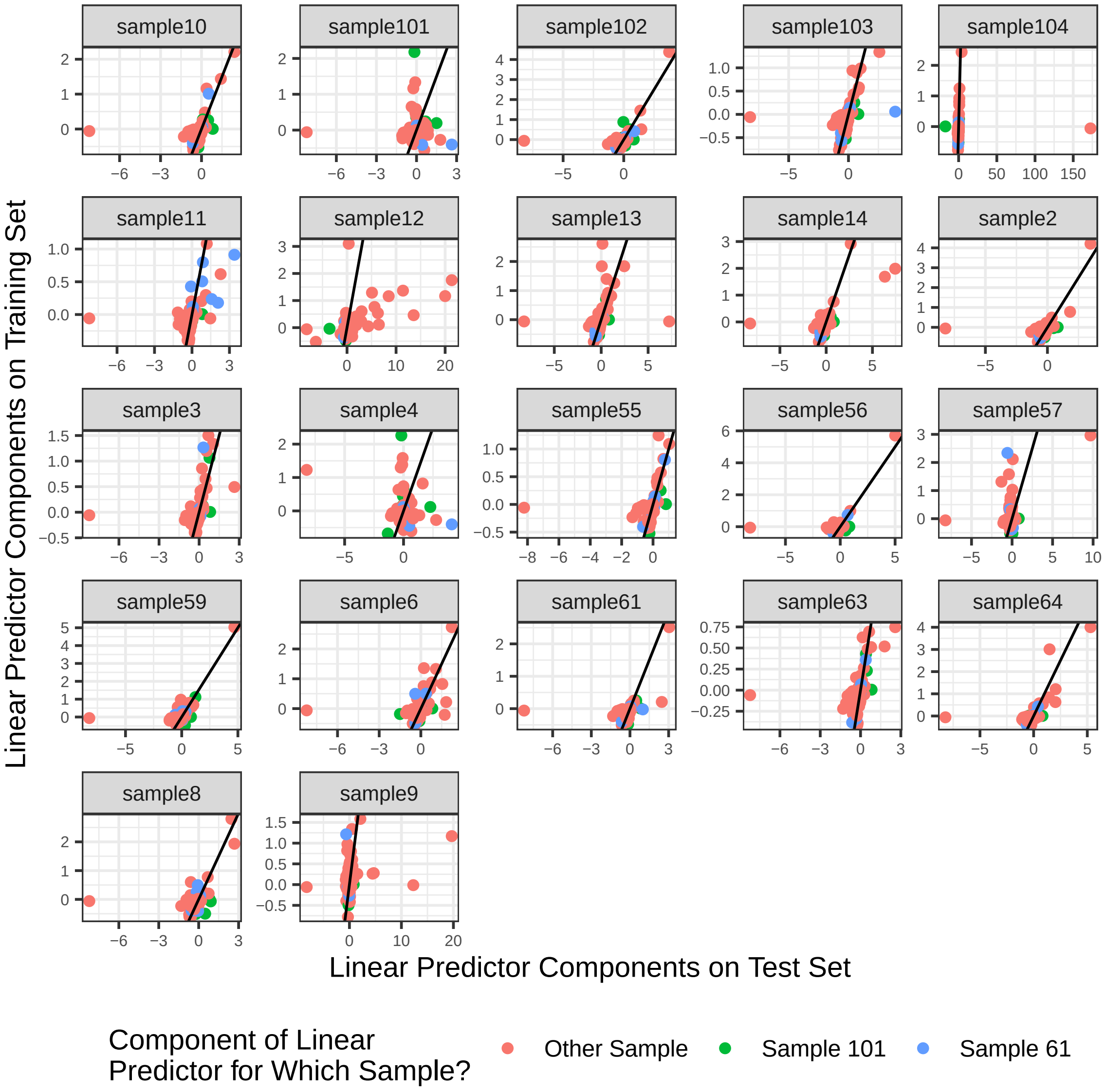

Fig. 7: Mean values of linear predictors (centered proportion multiplied by elastic net coefficients) by specimen for elastic net classifier trained on centered proportion genus-level specimen data. The line x = y is shown in black.

Web Appendix F: Bioinformatics Sensitivity Analysis

To assess the influence of our choice to treat results reported from different bioinformatics laboratories on individual samples sequenced by a single sequencing laboratory as replicate measurements, for each bioinformatics laboratory we included in our analysis, we fit and predicted from elastic net classifiers (classifying specimen) using only data reported by that laboratory. We limited this analysis to sequencing laboratories HL-B, HL-C, HL-F, HL-H, and HL-J, as other laboratories did not sequence enough samples to train and test elastic net classifiers on the basis of only one set of bioinformatics results.

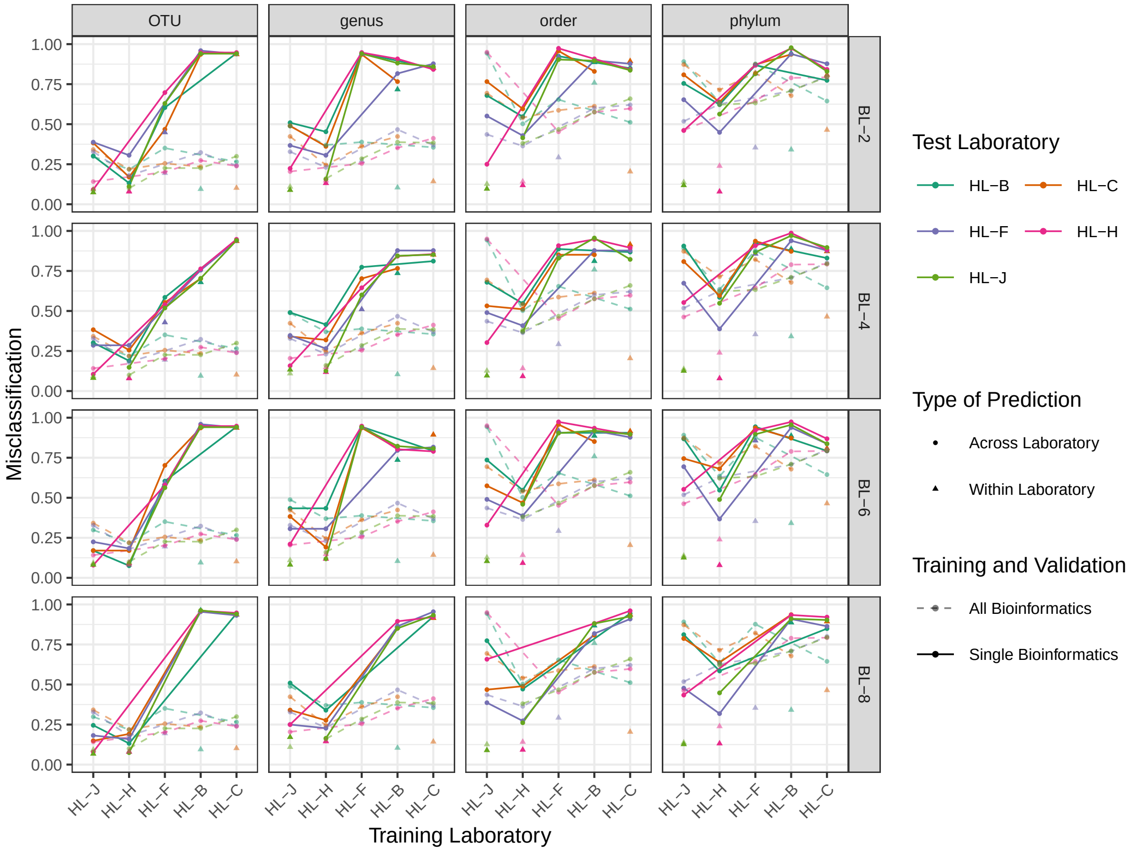

Fig. 8: Test set misclassification error for elastic net classifiers fit and validated on centered proportion data provided by individual bioinformatics laboratories (rows) is shown in bold against misclassification error for elastic net classifiers fit and validated on all four included bioinformatics laboratories, which is indicated by transparent points and lines. Performance of classifiers predicting within-sequencing-laboratory is indicated by triangles; cross-laboratory performance is shown with connected points.

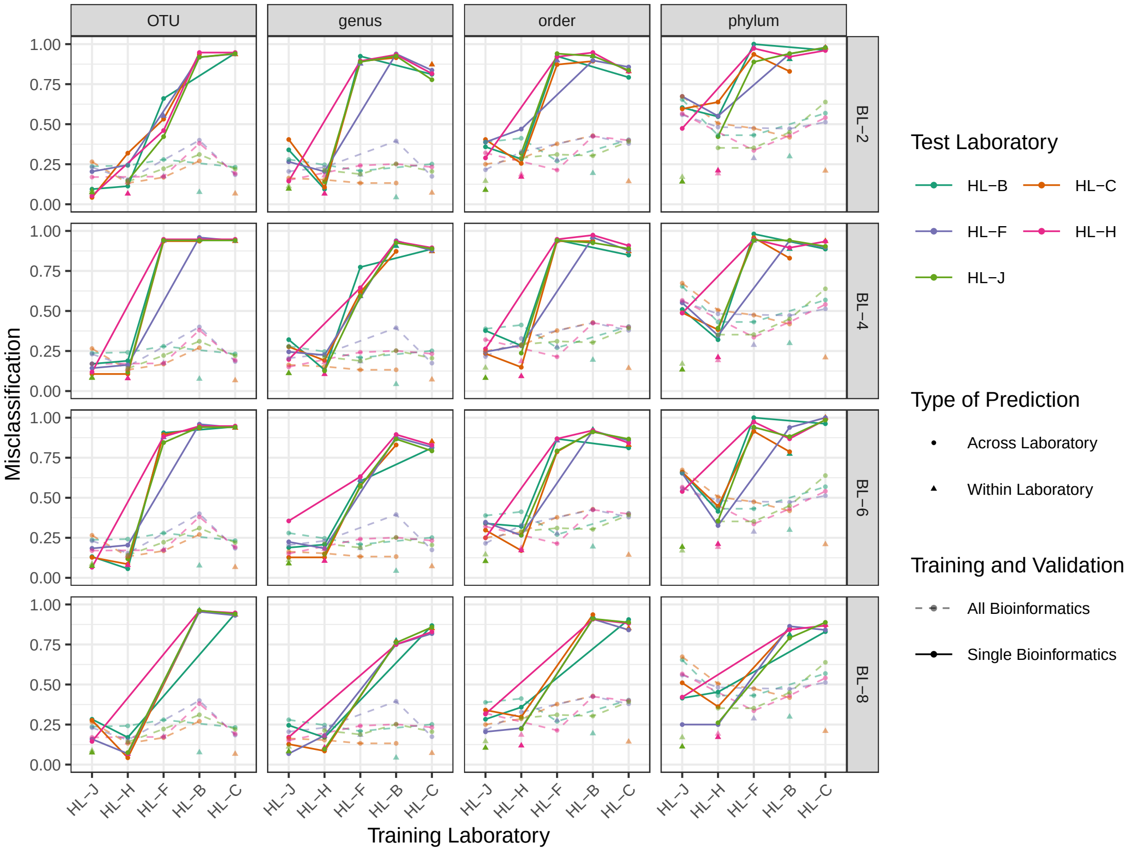

Fig. 9: Test set misclassification for error elastic net classifiers fit and validated on centered CLR data provided by individual bioinformatics laboratories (rows) is shown in bold against misclassification error for elastic net classifiers fit and validated on all four included bioinformatics laboratories, which is indicated by transparent points and lines. Performance of classifiers predicting within-sequencing-laboratory is indicated by triangles; cross-laboratory performance is shown with connected points.

For the two sequencing laboratories that processed the largest number of samples from sample types included in our analysis, HL-H (159 samples) and HL-J (265 samples), we are able to fit elastic net classifiers using data from single bioinformatics laboratories that distinguish (within sequencing laboratory) between specimens about as well as classifiers trained on all bioinformatics laboratories. We also observe largely similar patterns of within- versus across-sequencing laboratory misclassification for classifiers trained on individual bioinformatics data from HL-H

or HL-J as we do for classifiers trained on data from all bioinformatics laboratories for HL-J or HL-H.

For sequencing laboratories that processed fewer samples – HL-B (106 samples), HL-C (97 samples), HL-F (101 samples) – we are in general unable, on the basis of data from individual bioinformatics laboratories, to fit classifiers that distinguish between specimens within-laboratory. This renders comparison of cross-laboratory performance of these classifiers with cross-laboratory performance of classifiers fit on data from all bioinformatics laboratories difficult to interpret.

Web Appendix G: Classifier Performance under Centering and Scaling; Descriptive Analysis of Fresh Samples at Phylum Level

To explore whether taxon- and (sequencing) laboratory-specific scaling could explain our main results, we fit and predicted from elastic net classifiers using proportion- and centered log-ratio-transformed data to which we applied a sample centering (as described in section 3 of the manuscript) as well as a sample scaling. Under each transformation (proportion or CLR), in each test or training set, and for each taxon, we calculated scaling factors as the standard deviation of the mean observed value in each specimen (i.e., for each taxon we computed specimen means and calculated the standard deviation of these means). We then, after centering data, rescaled data by dividing by these taxon-specific scalings. We did not rescale taxa for which the computed scaling factor was 0. After applying this rescaling to each test and training set, we fit elastic net classifiers to identify specimen as in our primary analysis. The results of this analysis are shown in the figure below.

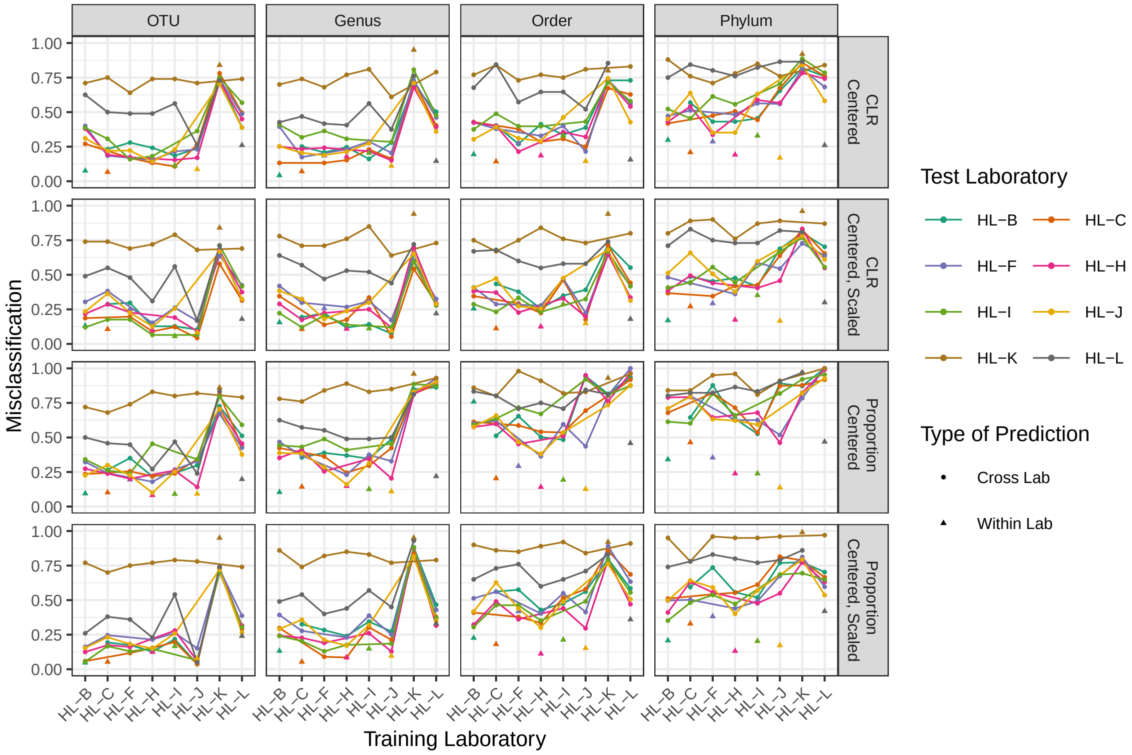

Fig. 10: Test set misclassification error for elastic net classifiers fit and validated on CLR (first two rows) and proportion data (third and fourth rows). For each transformation (CLR or proportion), we consider performance under sample centering (first and third rows) and under sample centering and scaling (second and fourth rows). Within-laboratory misclassification is indicated by triangles, and cross-laboratory misclassification is indicated by connected dots.

The comparison between within-laboratory and cross-laboratory misclassification is quite similar for centered and centered-and-scaled CLR data, with perhaps marginally better performance for classifiers fit and validated on scaled data than on unscaled genus data.

For proportion data, we observe greater improvement in cross-laboratory as compared to within-laboratory misclassification for classifiers fit on scaled data relative to those fit on unscaled data. This may reflect the fact that on the proportion scale, over- (or under-) detection of an abundant taxon results in a compression (or stretching) of measured proportions in other taxa (on account of the sum-to-one constraint). However, while the gap between within- and

cross-laboratory performance narrows for centered and scaled proportion data as compared to centered proportion data, cross-laboratory misclassification remains substantially higher than within-laboratory misclassification.

Descriptive Analyses

In order to provide intuition for the kinds of between-sequencing-laboratory differences we observe in between-specimen structure, we provide a descriptive analysis of abundances in fresh human samples for each of the four phyla (Firmicutes, Bacteroidetes, Actinobacteria, and Proteobacteria) directly analyzed by Sinha *and others* (2017). The following plots show measured abundances on the proportion and centered log-ratio scales in each of these phyla by sequencing laboratory, bioinformatics laboratory, health status, and specimen.

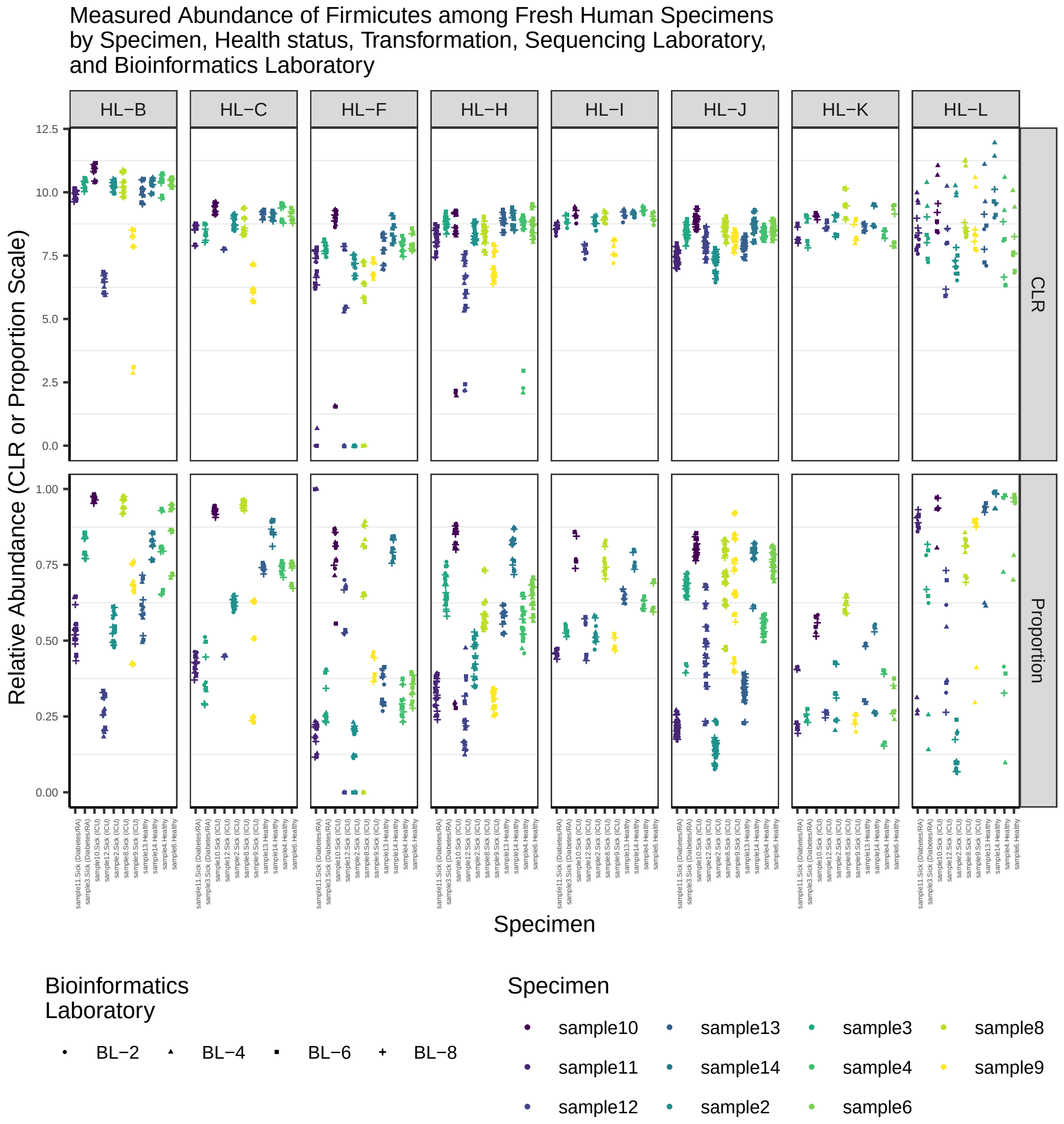

Fig. 11: Measured centered log-ratio (first row) and proportion (second row) Firmicutes abundance by sequencing laboratory (columns), bioinformatics laboratory (point shape), health status (x-axis), and specimen (color).

We observe, on both proportion and centered log-ratio scales, some shared between-specimen structure across sequencing laboratory. However, we also observe substantial qualitative differences across sequencing laboratory. For instance, is the centered log-ratio abundance of Firmicutes higher in specimen 13 (healthy; 4th from right) than in specimen 9 (ICU; 5th from right)? Measurements from HL-B and HL-H suggest so; measurements from HL-J do not. On the proportion scale, we, for instance, may come to substantially different conclusions about how similar the abundance of Firmicutes is in the two diabetes/RA samples depending on sequencing laboratory – HL-C suggests essentially no difference, HL-F suggests perhaps a small difference, and HL-H and HL-J suggest substantial differences.

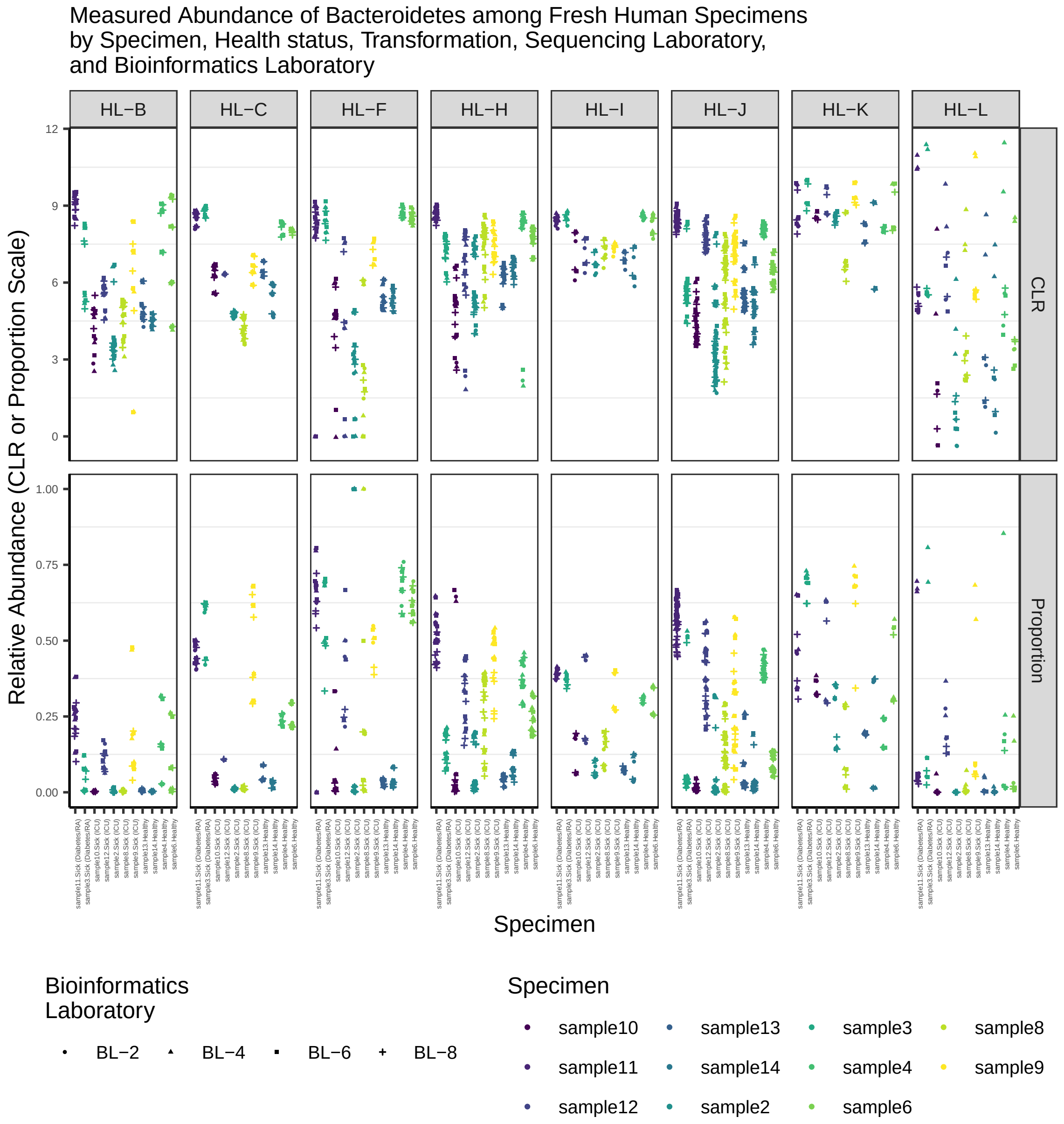

Fig. 12: Measured centered log-ratio (first row) and proportion (second row) Bacteroidetes abundance by sequencing laboratory (columns), bioinformatics laboratory (point shape), health status (x-axis), and specimen (color).

In terms of measured abundance of Bacteroidetes, we again observed partial concordance in terms of between-specimen structure across sequencing laboratories as well as examples of discordance. This is particularly evident at the proportion scale, where, for example, we might conclude that specimen 11 (first on left) has greater Bacteroidetes relative abundance than specimen 3 (second from left) if we consult HL-B or HL-H, whereas in HL-I we observe essentially no difference between these specimens. In a similar vein, determination of which specimen (among all specimens plotted) has highest relative abundance of Bacteroidetes differs across sequencing laboratory, with, notably, no consensus across laboratories regarding whether diabetes/RA (rheumatoid arthritis) specimens (at left) have generally higher or lower Bacteroidetes relative abundance than healthy specimens 4 and 6 (at right).

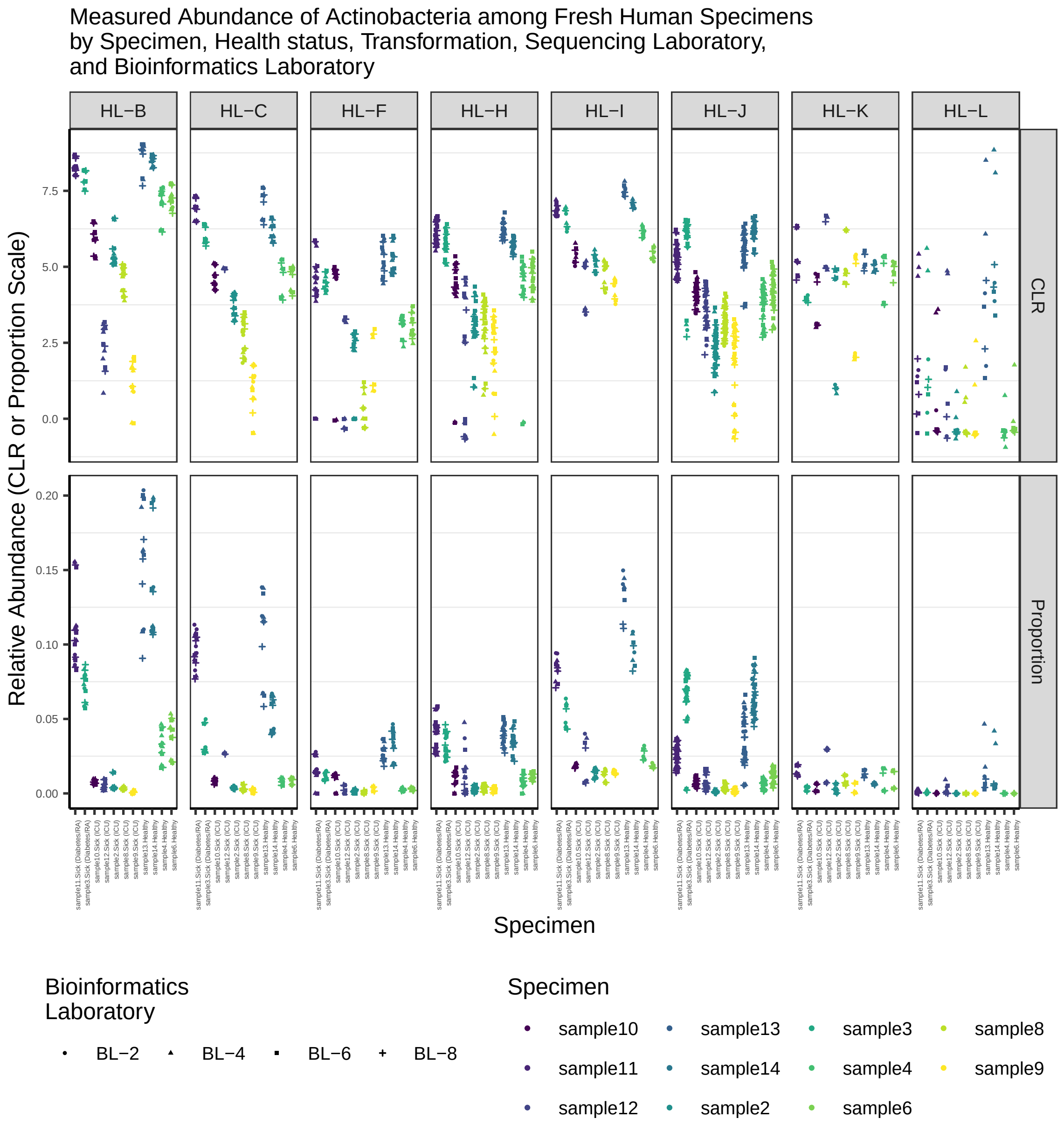

Fig. 13: Measured centered log-ratio (first row) and proportion (second row) Actinobacteria abundance by sequencing laboratory (columns), bioinformatics laboratory (point shape), health status (x-axis), and specimen (color).

We observe generally similar patterns in measured Actinobacteria abundance as we do for

Firmicutes and Bacteroidetes. In particular, some comparisons on the proportion scale replicate less well across sequencing laboratory on the proportion than on the centered log-ratio scale. For example, how do specimens 13 and 14 (healthy; 3rd and 4th from right) compare to specimens 11 and 3 (diabetes/RA; 1st and 2nd from left)? Is relative abundance (i.e., proportion) Actinobacteria higher in sample 11 or sample 3?

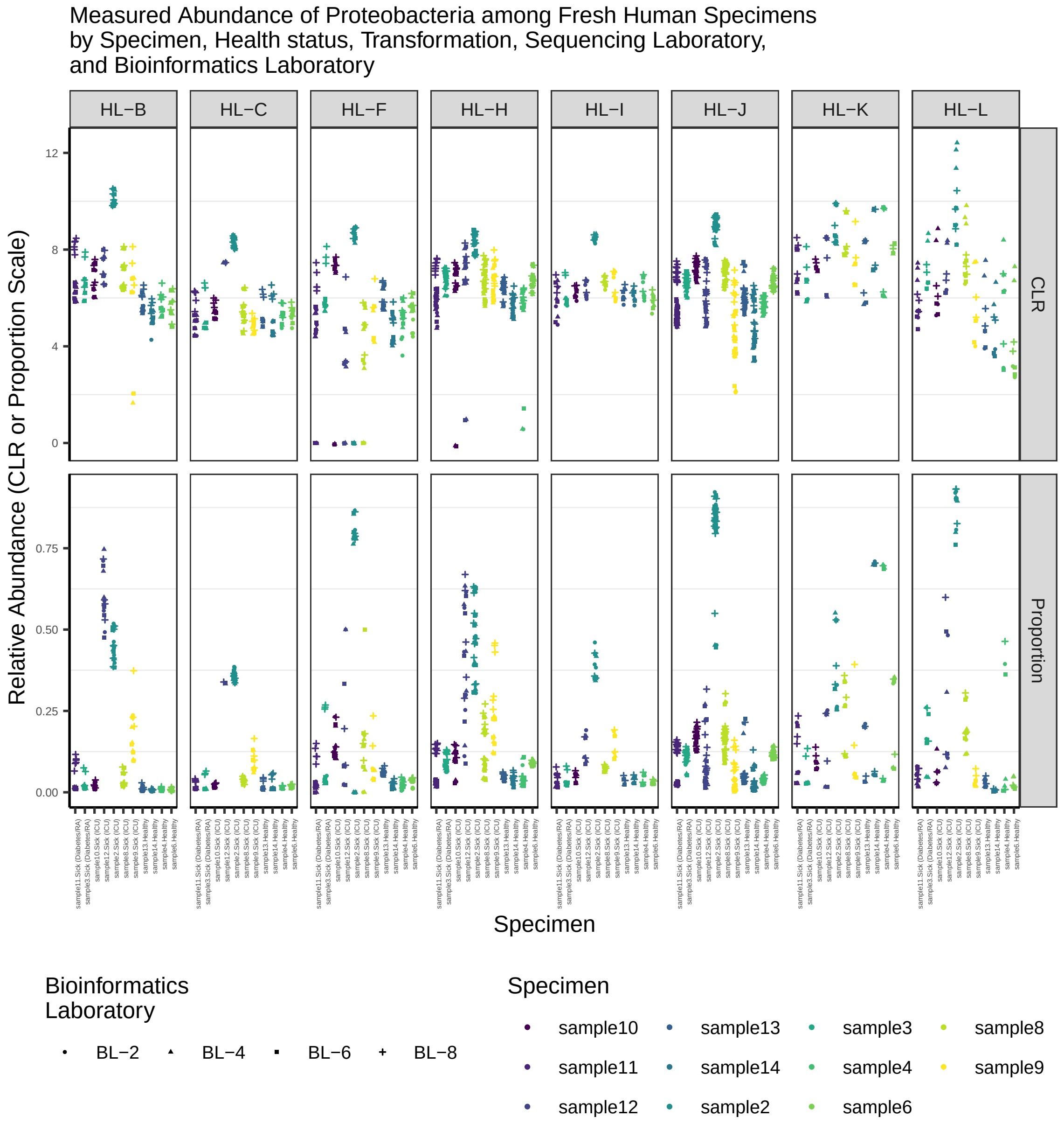

Fig. 14: Measured centered log-ratio (first row) and proportion (second row) Proteobacteria abundance by sequencing laboratory (columns), bioinformatics laboratory (point shape), health status (x-axis), and specimen (color).

On the centered log-ratio scale, all sequencing laboratories agree that specimen 2 (ICU; 5th

from left) has highest abundance Proteobacteria, though the degree to which this abundance exceeds abundances in other specimens differs somewhat by sequencing laboratory. On the proportion scale, the picture is not so clear – we might judge specimen 12 to have higher relative abundance Proteobacteria than specimen 2 on the basis of measurements from HL-B, and sequencing laboratories, furthermore, do not agree regarding whether specimen 12 has higher relative abundance Proteobacteria than specimens 8 and 9. On the centered log-ratio scale, HL-B suggests somewhat elevated Proteobacteria in diabetes/RA specimens (1st and 2nd from left) as compared to healthy specimens (rightmost four), but this distinction essentially disappears in HL-C and HL-I.

1. Web Appendix H: Classification on Presence Data

To assess the replicability of between-specimen signals observed under a commonly-used transformation of sequencing count data, the presence-absence transformation, we fit and predicted from classifiers using data at this scale. Concretely, we define the presence-absence transformation as follows:

$$\Psi: \mathbb{R}^J \rightarrow \{0,1\}^J; \Psi(\vec{W}_{i\cdot k}) = \left(\mathbf{1}_{[W_{i1k}>0]}, \ldots, \mathbf{1}_{[W_{iJk}>0]}\right).$$

Consideration of 16S data on the presence-absence scale was motivated by the practice of treating 16S amplicon data as non-quantitative in the sense measured proportions of 16S variants do not reflect the true proportions of the taxa to which they are assigned (Méheust *and others*, 2019; Kennedy *and others*, 2014; Costa *and others*, 2012). Hence, this line of reasoning suggests, it may be more appropriate to treat 16S amplicon data as indicating only presence or absence of taxa. That is, in the presence of measurement error, between-specimen comparisons based on presence-absence data should be either invariant or at least less variable than proportion-scale comparisons.

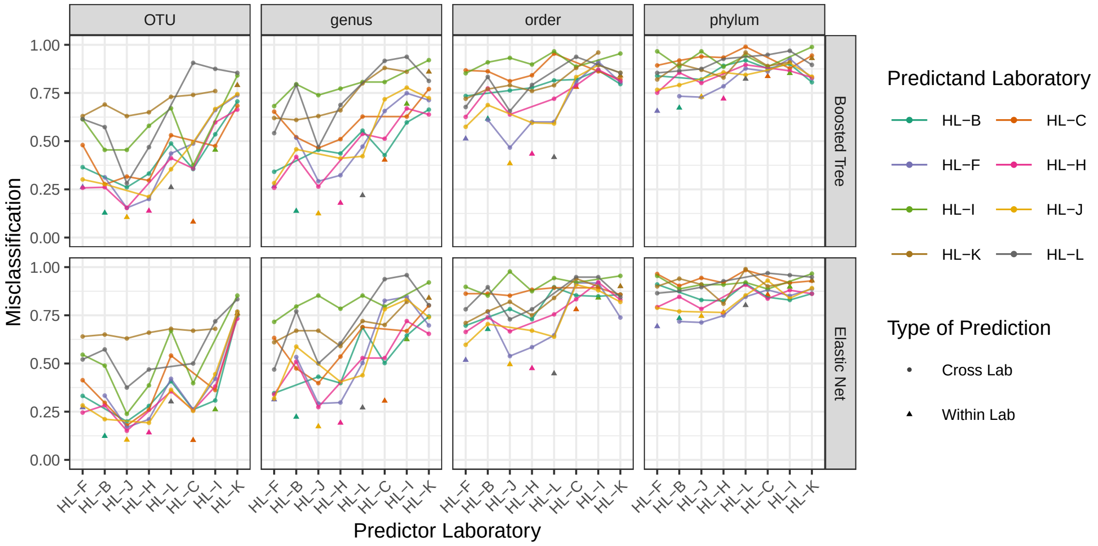

Fig. 15: Within-laboratory (solid triangles) and between-laboratory (lines) misclassification for boosted tree and elastic net classifiers predicting specimen on presence data plotted against level of taxonomic aggregation.

We chose not to center presence-absence data, as this would produce continuous data, and we were interested in the consistency of binary measurements across laboratories. Hence, the error model presented in Section 2.2 (main text) is not directly relevant here. The results in this section therefore pertain to whether observed patterns of differential presence of taxa across specimens are likely to replicate across different sequencing laboratories.

At every level of taxonomic aggregation, and for both elastic net and boosted tree classification, median cross-laboratory misclassification of specimen classifiers is larger than median within-laboratory misclassification (Figure 15). Both within- and cross-laboratory misclassification increase with increasing level of taxonomic aggregation. On OTU-level data, median within-laboratory misclassification of boosted tree classifiers is 20% (IQR 12% - 31%), versus 49% (IQR 33% - 66%) median cross-laboratory misclassification. On phylum data, these figures are 78% (IQR 71% - 84%) and 88% (IQR 84% - 92%), respectively. Similar patterns hold for elastic net classifiers, with 27% (IQR 14% - 39%) and 46% (IQR 28% - 68%) within- and cross-laboratory

misclassification on OTU-level data, rising to 78% (IQR 74% - 87%) and 89% (IQR 84% - 92%), respectively, on phylum data.

2. Web Appendix I: Comparison Across Transformations

This section contains within- and cross-laboratory misclassification results for all levels of taxonomic aggregation, as well as results on data that has not been sample-centered.

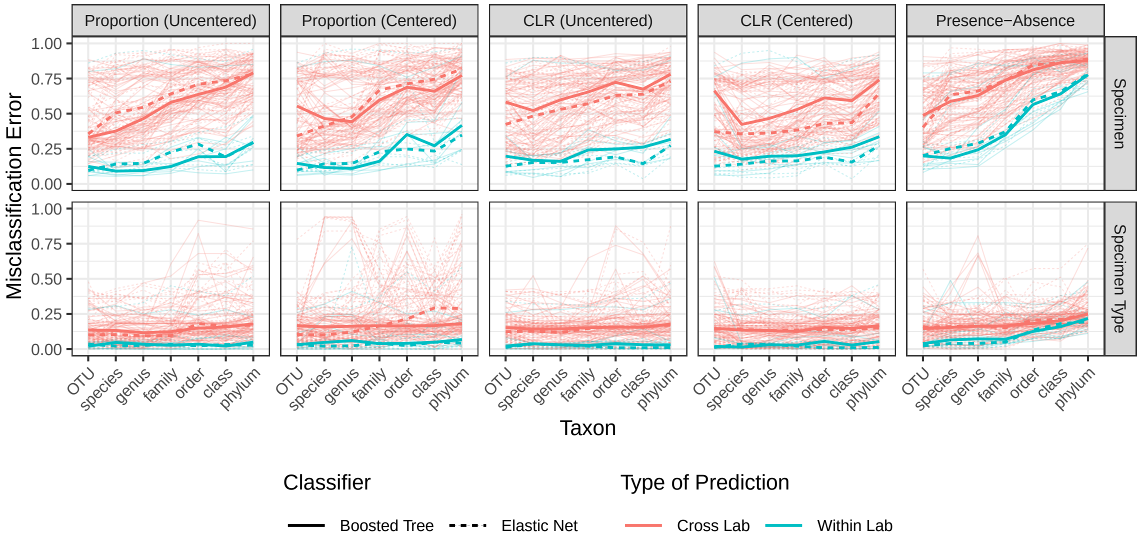

Fig. 16: The within (aqua) and across (red) laboratory misclassification for uncentered proportions, centered proportions, uncentered log ratio, centered log ratio and presence absence data for both classifying both specimen and specimen type. The misclassification rate is shown for boosted tree (solid lines) and elastic net (dotted lines) classifiers for every combination of laboratories (thin lines) and is also summarized as a median across laboratory combinations (thick lines). We see that centering the centered log ratio transformation improves the misclassification rate, but centering the proportions does not improve the misclassification rate.

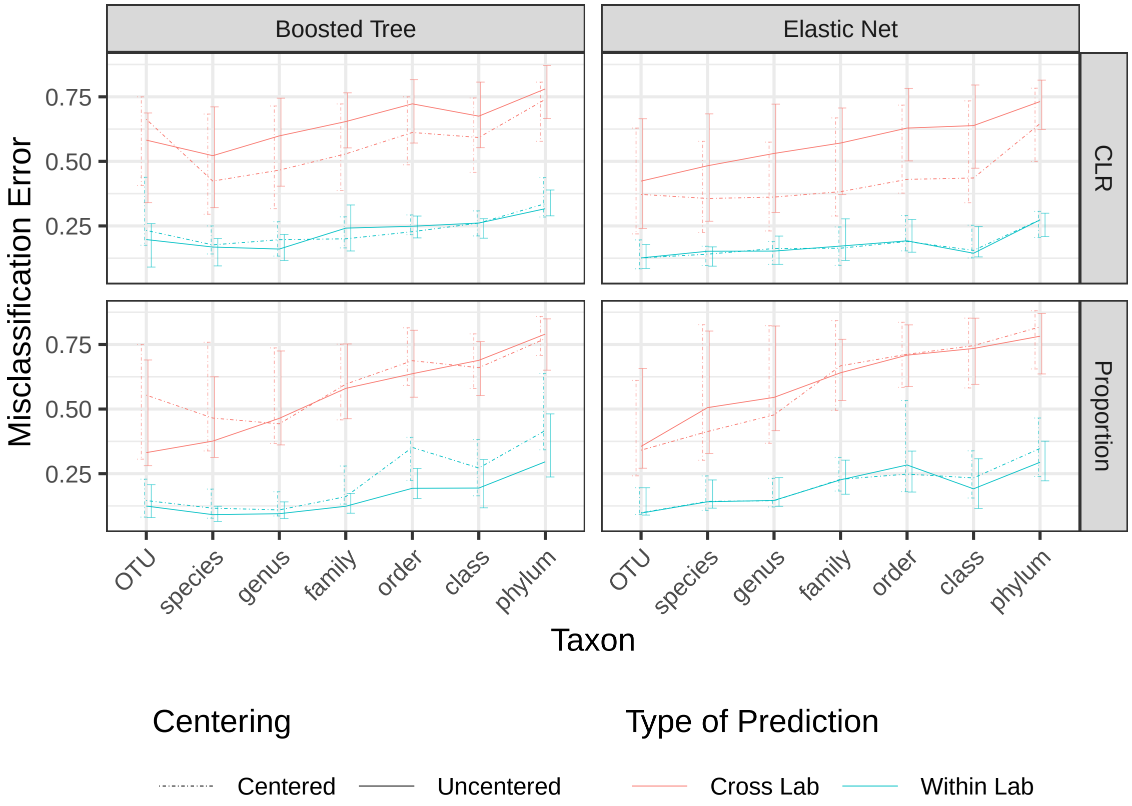

Fig. 17: Median within-laboratory (aqua) and cross-laboratory (red) specimen misclassification for proportion and centered-log-ratio data, with and without sample centering. The interquartile range is shown in brackets at each taxon.

Results for Uncentered Proportion Data

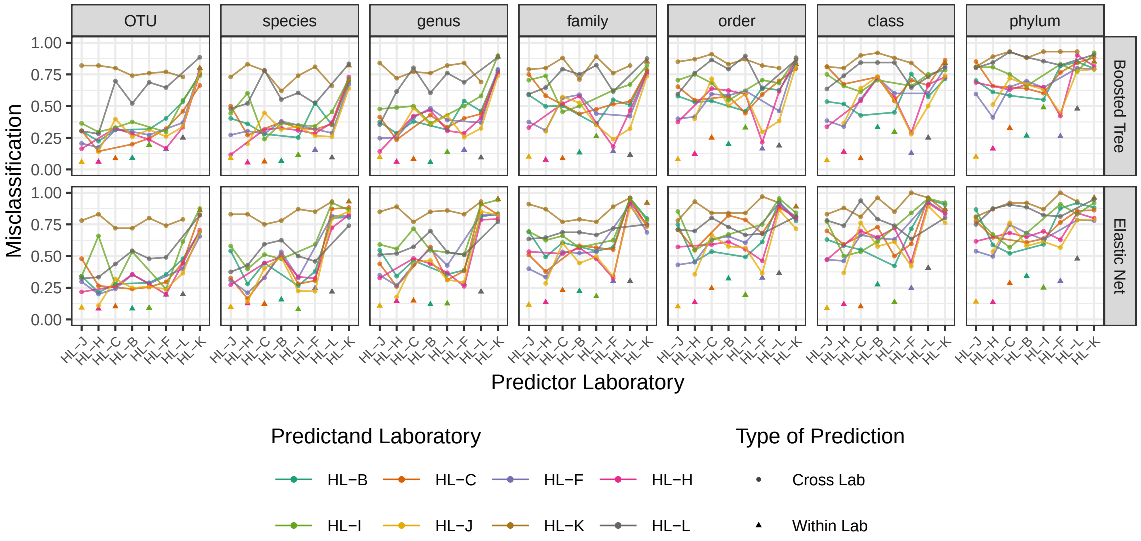

Fig. 18: Within-laboratory (solid triangles) and between-laboratory (lines) specimen misclassification results on uncentered proportion data.

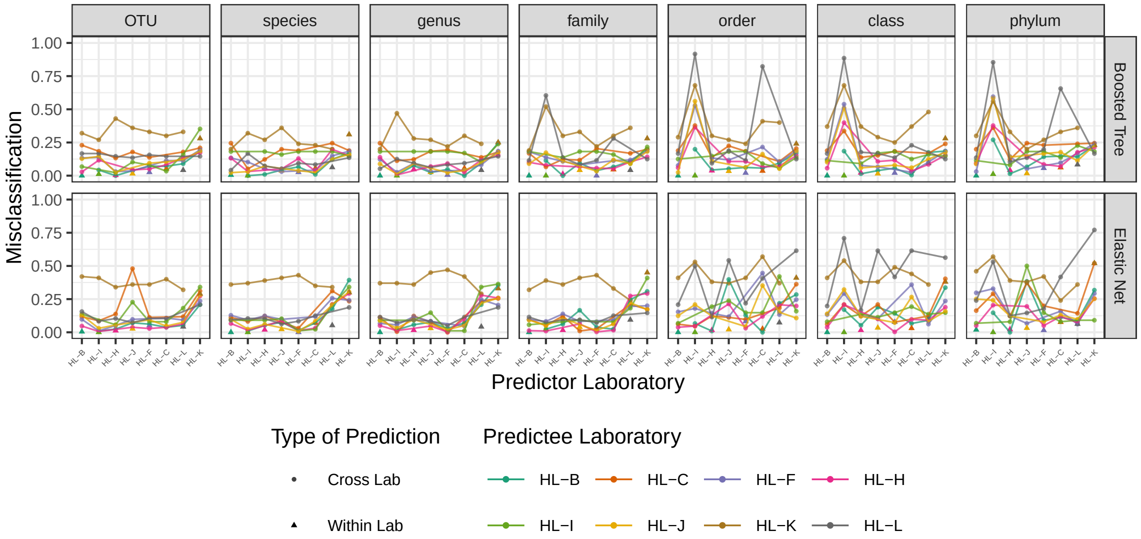

Fig. 19: Within-laboratory (solid triangles) and between-laboratory (lines) specimen type misclassification results on uncentered proportion data.

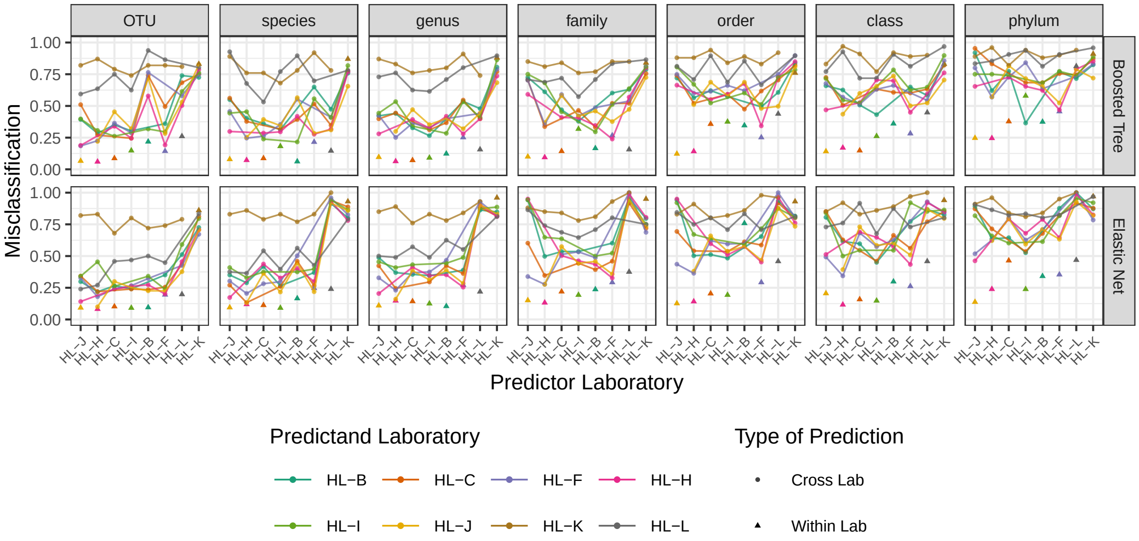

Fig. 20: Within-laboratory (solid triangles) and between-laboratory (lines) specimen misclassification results on centered proportion data.

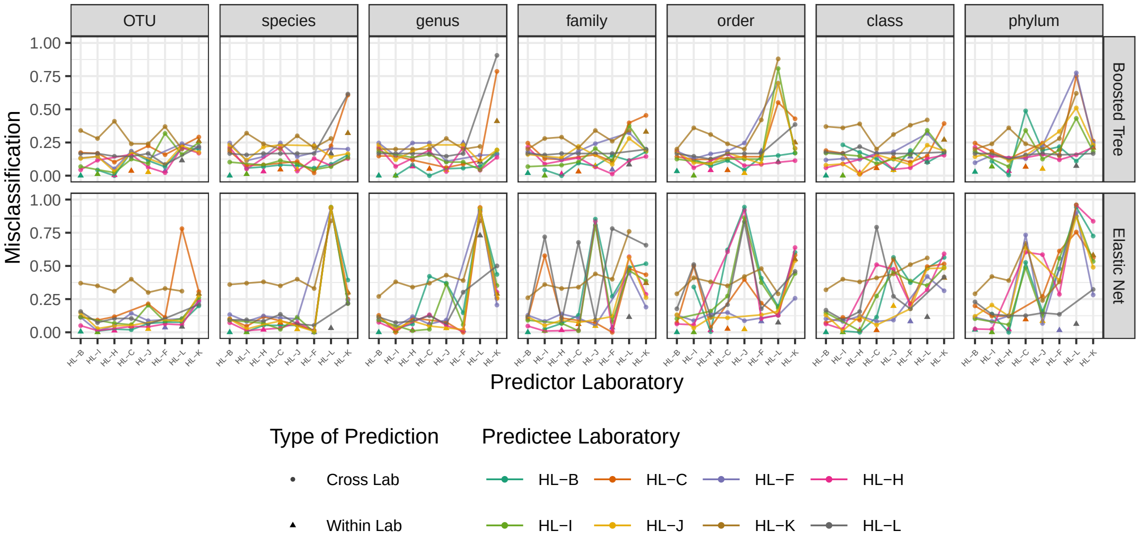

Fig. 21: Within-laboratory (solid triangles) and between-laboratory (lines) specimen type misclassification results on centered proportion data.

Results for Uncentered Log-Ratio Data

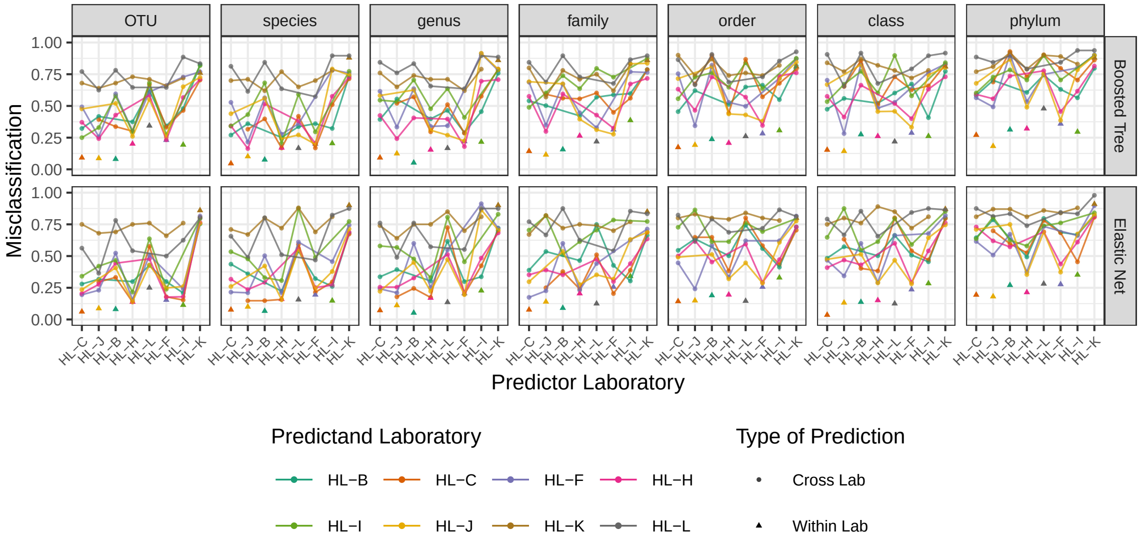

Fig. 22: Within-laboratory (solid triangles) and between-laboratory (lines) specimen misclassification results on log-ratio data (without sample centering).

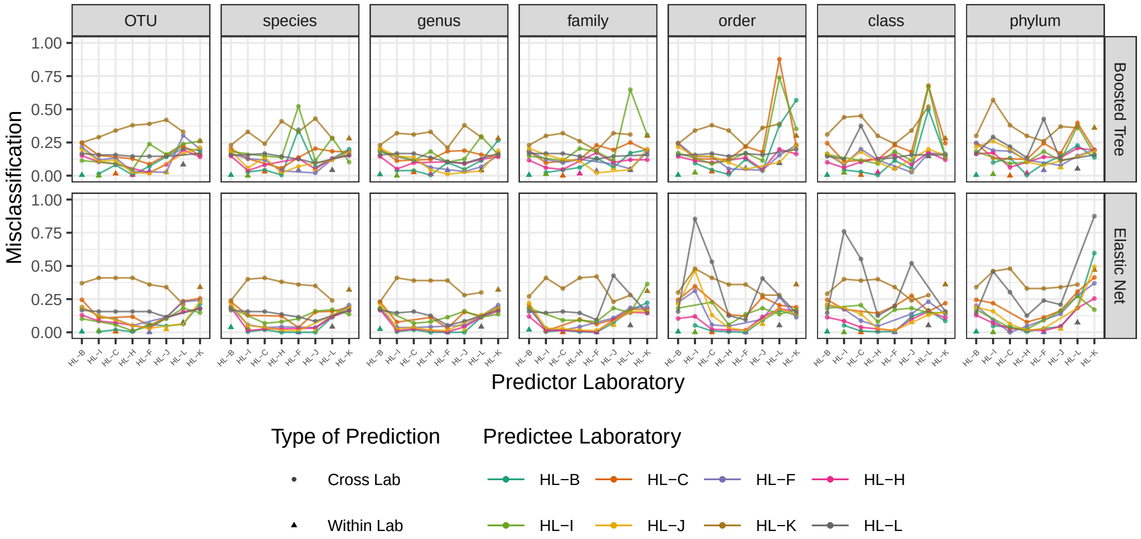

Fig. 23: Within-laboratory (solid triangles) and between-laboratory (lines) specimen type misclassification results on log-ratio data (without sample centering).

Results for Centered Log-Ratio-Scale Data

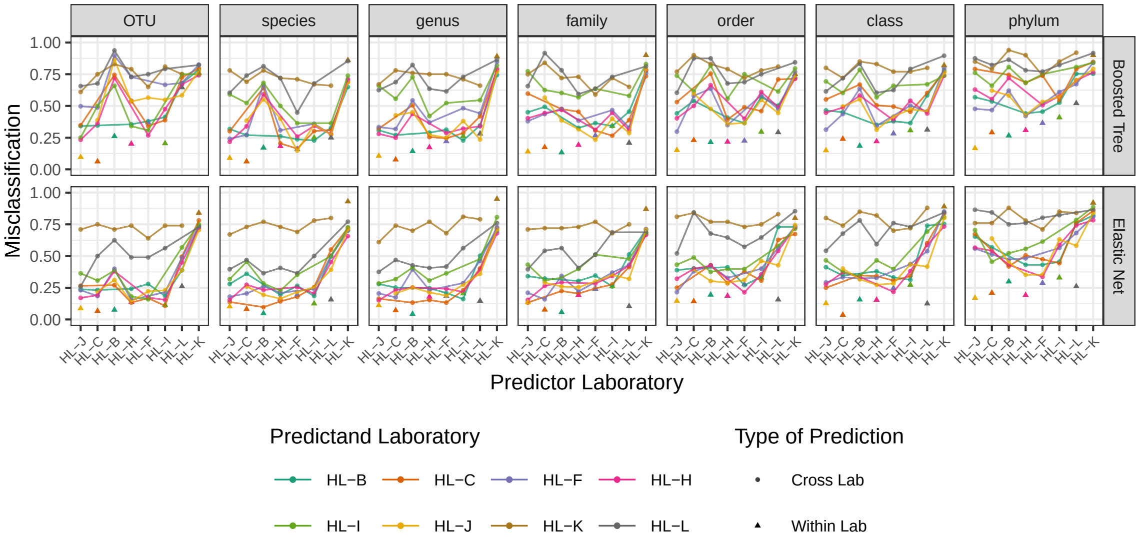

Fig. 24: Within-laboratory (solid triangles) and between-laboratory (lines) specimen misclassification results on log-ratio data (with sample centering).

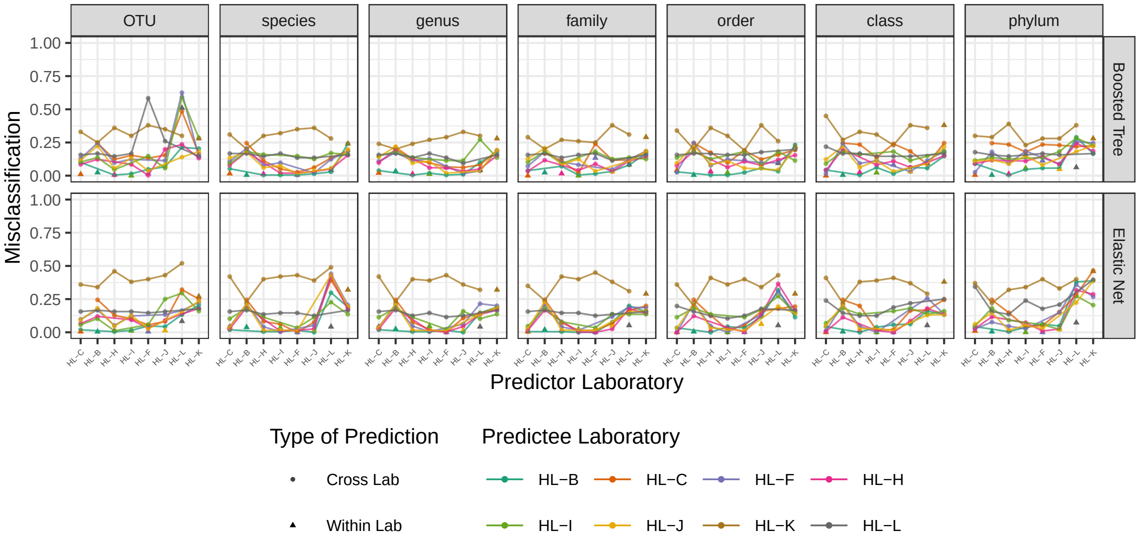

Fig. 25: Within-laboratory (solid triangles) and between-laboratory (lines) specimen type misclassification results on log-ratio data (with sample centering).

Results for Presence-Absence Data

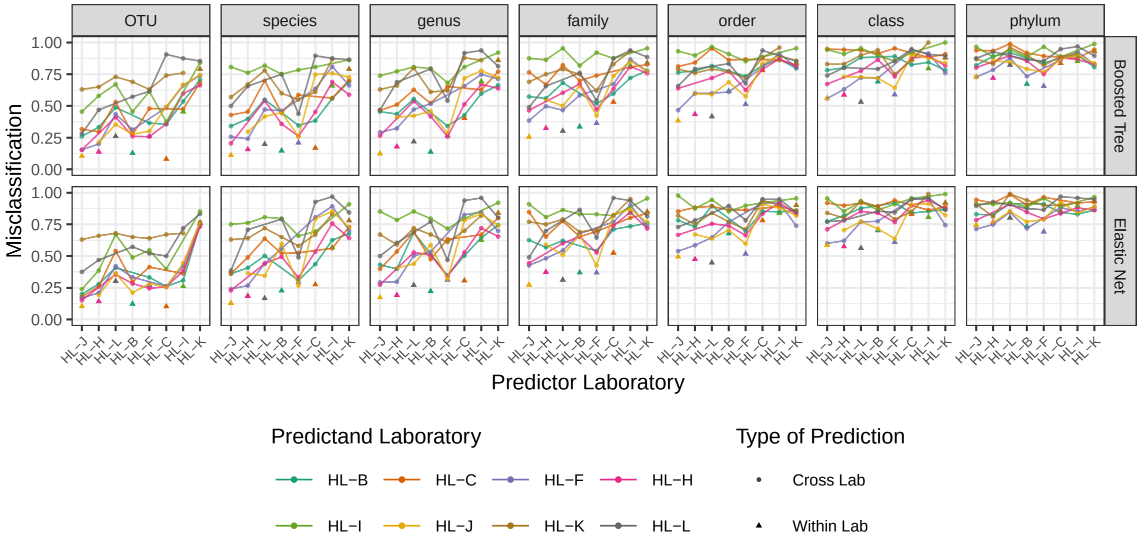

Fig. 26: Within-laboratory (solid triangles) and between-laboratory (lines) specimen misclassification results on presence-absence data (without sample centering).

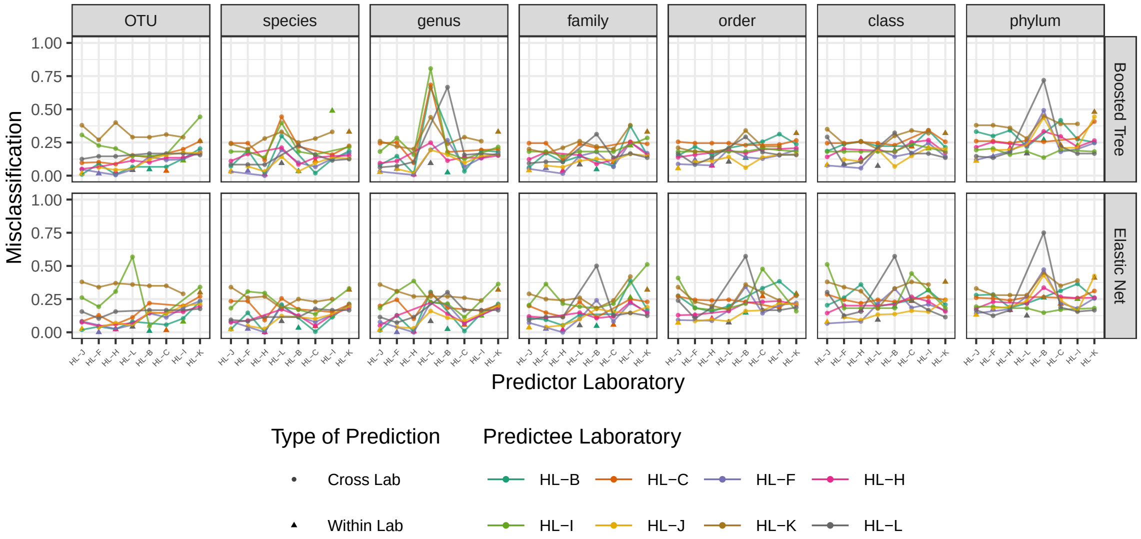

Fig. 27: Within-laboratory (solid triangles) and between-laboratory (lines) specimen type misclassification results on presence-absence data (without sample centering).

References

Chen, Tianqi, He, Tong, Benesty, Michael, Khotilovich, Vadim, Tang, Yuan, Cho, Hyunsu, Chen, Kailong, Mitchell, Rory, Cano, Ignacio, Zhou, Tianyi, Li, Mu, Xie, Junyuan, Lin, Min, Geng, Yifeng *and others.* (2019). *xgboost: Extreme Gradient Boosting*. R package version 0.90.0.2.

Costa, Marcio C, Arroyo, Luis G, Allen-Vercoe, Emma, Stämpfli, Henry R, Kim, Peter T, Sturgeon, Amy and Weese, J Scott. (2012). Comparison of the fecal microbiota of healthy horses and horses with colitis by high throughput sequencing of the V3-V5 region of the 16s rRNA gene. *PLOS ONE* **7**(7), e41484.

Elith, Jane, Leathwick, John R and Hastie, Trevor. (2008). A working guide to boosted regression trees. *Journal of Animal Ecology* **77**(4), 802–813.

Friedman, Jerome, Hastie, Trevor and Tibshirani, Rob. (2010). Regularization paths for generalized linear models via coordinate descent. *Journal of Statistical Software* **33**(1), 1.

Kennedy, Nicholas A, Walker, Alan W, Berry, Susan H, Duncan, Sylvia H, Farquarson, Freda M, Louis, Petra and Thomson, John M. (2014). The impact of different dna extraction kits and laboratories upon the assessment of human gut microbiota composition by 16s rRNA gene sequencing. *PLOS ONE* **9**(2), e88982.

McLaren, Michael R, Willis, Amy D and Callahan, Benjamin J. (2019). Consistent and correctable bias in metagenomic sequencing experiments. *eLife* **8**.

Méheust, Raphaël, Burstein, David, Castelle, Cindy J and Banfield, Jillian F. (2019). The distinction of CPR bacteria from other bacteria based on protein family content. *Nature Communications* **10**(1), 1–12.

Sinha, Rashmi, Abu-Ali, Galeb, Vogtmann, Emily, Fodor, Anthony A, Ren, Boyu, Amir, Amnon, Schwager, Emma, Crabtree, Jonathan, Ma, Siyuan, Abnet, Christian C, Knight, Rob, White, Owen *and others*. (2017). Assessment of variation in microbial community amplicon sequencing by the Microbiome Quality Control (MBQC) project consortium. *Nature Biotechnology* **486**(11), 207.

Vebø, Heidi C, Karlsson, Magdalena Kauczynska, Avershina, Ekaterina, Finnby, Lene and Rudi, Knut. (2016). Bead-beating artefacts in the bacteroidetes to firmicutes ratio of the human stool metagenome. *Journal of Microbiological Methods* **129**, 78–80.
